# New Investigations with Lupane Type A-Ring Azepane Triterpenoids for Antimycobacterial Drug Candidate Design

**DOI:** 10.3390/ijms222212542

**Published:** 2021-11-21

**Authors:** Oxana Kazakova, Roxana Racoviceanu, Anastasiya Petrova, Marius Mioc, Adrian Militaru, Lucreția Udrescu, Mihai Udrescu, Adrian Voicu, Jason Cummings, Gregory Robertson, Diane J. Ordway, Richard A. Slayden, Codruța Șoica

**Affiliations:** 1 Ufa Institute of Chemistry, The Ufa Federal Research Centre, The Russian Academy of Sciences, 71, Pr. Oktyabrya, 450054 Ufa, Russia; ana.orgchem@gmail.com; 2 Department II-Pharmaceutical Chemistry, Faculty of Pharmacy, “Victor Babeş” University of Medicine and Pharmacy Timişoara, 2 Eftimie Murgu Sq., 300041 Timişoara, Romania; babuta.roxana@umft.ro (R.R.); marius.mioc@umft.ro (M.M.); codrutasoica@umft.ro (C.Ș.); 3 Res Ctr Pharmacotoxicol Evaluat, Faculty of Pharmacy, “Victor Babeş” University of Medicine and Pharmacy Timisoara, Eftimie Murgu Sq. 2, 300041 Timisoara, Romania; 4 Department of Computer and Information Technology, University Politehnica of Timişoara, 2 Vasile Pârvan Blvd., 300223 Timişoara, Romania; militaru.adrian25@gmail.com (A.M.); mudrescu@cs.upt.ro (M.U.); 5 Department I-Drug Analysis, Faculty of Pharmacy, “Victor Babeş” University of Medicine and Pharmacy Timişoara, 2 Eftimie Murgu Sq., 300041 Timişoara, Romania; udrescu.lucretia@umft.ro; 6 Department III-Informatics and Medical Biostatistics, Faculty of Pharmacy, “Victor Babeş” University of Medicine and Pharmacy Timişoara, 2 Eftimie Murgu Sq., 300041 Timişoara, Romania; 7 Department of Microbiology, Immunology & Pathology, Colorado State University, 1619 Campus Delivery, Fort Collins, CO 80523, USA; j.cummings@colostate.edu (J.C.); gregory.robertson@colostate.edu (G.R.); d.ordway@colostate.edu (D.J.O.); richard.slayden@colostate.edu (R.A.S.)

**Keywords:** triterpenoids, lupane, betulin, azepane, *M. tuberculosis*, resistant MTB strains, *M. abscessus*, *M. avium*, cytotoxicity

## Abstract

Twenty lupane type A-ring azepano-triterpenoids were synthesized from betulin and its related derivatives and their antitubercular activity against *Mycobacterium tuberculosis*, mono-resistant MTB strains, and nontuberculous strains *Mycobacterium abscessus* and Mycobacterium *avium* were investigated in the framework of AToMIc (Anti-mycobacterial Target or Mechanism Identification Contract) realized by the Division of Microbiology and Infectious Diseases, NIAID, National Institute of Health. Of all the tested triterpenoids, 17 compounds showed antitubercular activity and 6 compounds were highly active on the H37Rv wild strain (with MIC 0.5 µM for compound **7**), out of which 4 derivatives also emerged as highly active compounds on the three mono-resistant MTB strains. Molecular docking corroborated with a machine learning drug-drug similarity algorithm revealed that azepano-triterpenoids have a rifampicin-like antitubercular activity, with compound **7** scoring the highest as a potential *M. tuberculosis* RNAP potential inhibitor. FIC testing demonstrated an additive effect of compound **7** when combined with rifampin, isoniazid and ethambutol. Most compounds were highly active against *M. avium* with compound **14** recording the same MIC value as the control rifampicin (0.0625 µM). The antitubercular ex vivo effectiveness of the tested compounds on THP-1 infected macrophages is correlated with their increased cell permeability. The tested triterpenoids also exhibit low cytotoxicity and do not induce antibacterial resistance in MTB strains.

## 1. Introduction

Despite the remarkable progress made in the 20th century in terms of prevention and therapeutic approaches, tuberculosis (TB) still remains one of the deadliest diseases worldwide, with devastating personal, social, and economic impacts. In light of these alarming aspects, the World Health Assembly adopted in 2014 the WHO’s post-2015 End TB Strategy which aims to reduce TB incidence, mortality, and treatment costs by 2030 and is based on three pillars, one of them being the intensification of research and innovation which is considered critical in stopping the TB epidemic evolution [1]. The theme of the World TB Day 2021, commemorated each year on March 24, was “The Clock is Ticking”, thus sounding an alarm signal that the fight against tuberculosis is not achieving the expected progress [2]. This is particularly true in the context of the COVID-19 pandemic, which diverted substantial financial and personnel resources to act against the new threat and thus put the End TB program at risk (and, in particular, in the most burdened countries). There is therefore an urgent need to develop new strategies in order to prevent and cure this ongoing threat to public health.

Lupane is one of the main chemical scaffolds found in pentacyclic triterpenes, which are secondary metabolites synthesized in superior plants and composed of 6 isoprene units and 30 carbon atoms [3]. The most thoroughly studied lupane-type pentacyclic triterpenes are betulin, betulinic acid, and lupeol, which were reported as strong chemopreventive and anticancer agents against a large variety of cancer cells [4,5,6,7,8]. However, these compounds exhibit a multitude of additional pharmacological activities, including antimycobacterial effects, which can be exploited in the efforts to fight TB infection [9,10,11]. Betulin was the subject of a patent as tuberculosis treating and preventing agent [12]. Also, other lupane triterpenes active against *M. tuberculosis* were identified in various plant species: betulinic acid methylenediol ester in *Syzygium guineense* Wild DC. (Myrtaceae) stem bark [13], two lupane derivatives in *Chrysanthemum morifolium* (Asteraceae) with selective toxicity on *M. tuberculosis* [14], 2-*O-E-p*-coumaroylalphitolic acid, and alphitolic acid in *Ziziphus cambodiana* [15], and betulone in *Alnus incana* [16].

The discovery of the complex pharmacological profile of lupane-type pentacyclic triterpenes has triggered numerous efforts to generate semisynthetic derivatives in order to improve their pharmacokinetic and pharmacodynamic parameters and thus to identify new lead compounds. However, most researchers focused on testing the cytotoxic and antiproliferative activity of such derivatives [7,17,18,19,20,21]. Fewer studies have assessed their antimycobacterial potential.

By using a molecular docking approach, 3-epi-betulinic acid acetate was reported as a strong inhibitor of rhamnose biosynthesis in the cell wall of *M. tuberculosis* [22]. The modulation of the C3-position in betulinic acid as cinnamate-based esters resulted in new compounds with antimycobacterial properties and allowed the establishment of structure-activity relationships [23]. Acetyl-betulinic acid was tested against *M. tuberculosis* H37Rv and proved more active compared to betulinic acid [24]. Conjugates of betulinic acid with isoniazid and 3- and 4-pyridinecarboxaldehydes revealed some promising results during preliminary assays [25]. Based on the observation that the antimycobacterial activity of triterpenoids depends on their lipophilicity, which allows their penetration through the cell wall as well as the fact that nitrogen-bearing compounds form the predominant part of active anti-tuberculosis agents, a series of azepano-triterpenoids as a novel class of antitubercular compounds have been synthesized [26]. The MIC values of compounds ranged from 3.125 to >200 µM and A-azepano-28-cinnamoyloxybetulin was revealed as a lead derivative with MIC 2 µM and MBC 4 µM against MTB H37Rv and MICs values from 1 to 4 µM against isoniazid-, rifampicin- and ofloxacin-resistant strains, respectively. It was also shown that the introduction of the azepane moiety into lupane scaffold as well as conjugation of triterpenoids with hydrazine hydrate or isoniazid at C3 or C28 positions improves the activity against *M. tuberculosis* [27]. In addition, azepanobetulin was reported as a lead compound against isoniazid-resistant strains. In light of these findings, azepanobetulin and its amide derivative were further investigated; in vitro studies under aerobic and anaerobic environment revealed strong antimycobacterial effects on the H37Rv MTB strain as well as non-tuberculous mycobacterial strains (*M. avium, M. abscessus*) [28]. Azepanobetulin also exhibited a suitable pharmacokinetic profile and was therefore submitted to in vivo experiments, which recorded a statistically significant antimycobacterial activity. Molecular docking studies showed that the antimycobacterial mechanism of action of azepano-triterpenoids might consist in the inhibition of tuberculosinyl adenosine transferase (Rv3378c) [28,29].

In order to further study the antitubercular activity of lupane type azepane derivatives, we used the possibilities of the Division of Microbiology and Infectious Diseases of the National Institutes of Allergy and Infectious Diseases (http://www.niaid-aacf.org/, accessed on 1 October 2019) and participated in the screening of a series compounds in the framework of “Anti-mycobacterial Target or Mechanism Identification Contract (AToMIc)”.

## 2. Results and Discussion

### 2.1. Chemistry

**Figure 1:**
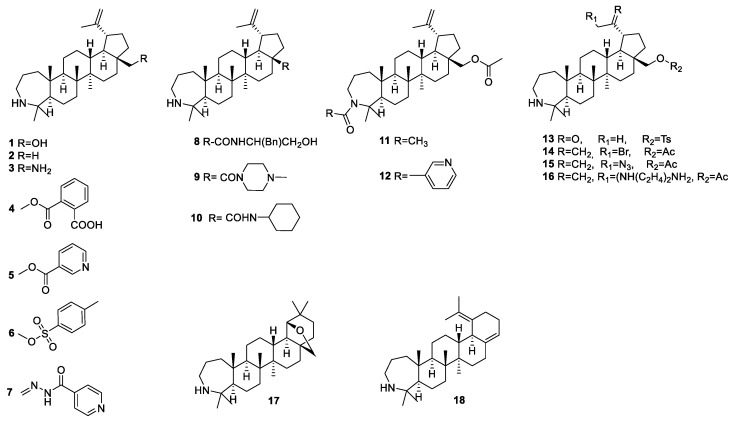
The structures of previously synthesized [26,29,30,31,32,33,34,35] lupane-type azepano-triterpenoids.

**Scheme 1:**
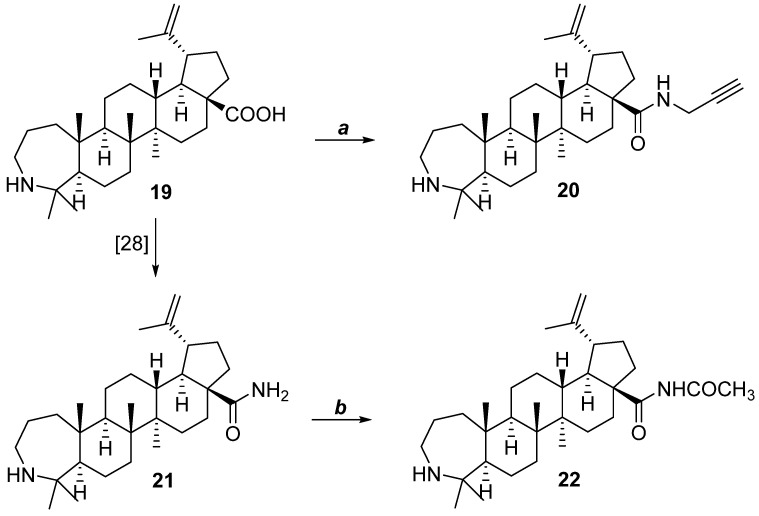
Synthesis of compounds **20** and **22**. a. i. (COCl)_2_, Et_3_N, CH_2_Cl_2_, 25 °C; ii. propargyl amine, Et_3_N, CH_2_Cl_2_, 40 °C, 2 h; b. glacial AcOH, reflux, 3 h.

In this study, a total of twenty lupane type A-ring azepano-triterpenoids were synthesized from betulin and its related derivatives according to methods described earlier [26,29,30,31,32,33,34,35]. Their structures are presented in Figure 1. The synthesis of two new amides is shown at Scheme 1. Firstly, azepanobetulinic acid **19** [27] was transformed to propargyl-amide **20** by the chloride method with a yield of 64%. Azepanobetulinic acid carboxamide **21** [28] was acylated by glacial AcOH under reflux to afford acetyl-derivative **22** in a yield of 79%. The structures and the purity of the synthesized compounds **20** and **22** were confirmed by elemental analysis, NMR spectroscopy, and mass-spectrometry. Thus, in ^13^C NMR spectra of compounds **20** and **22** the signals of carboxamide group C28 were observed at 176.5 and 170.6 ppm. The signals of the triple bond of compound **20** at 70.8 (CH) and 80.4 (C) were characteristic.

### 2.2. Machine Learning Antitubercular Activity and Compound-Drug Similarity Prediction

Machine learning techniques aim at finding subtle correlations between InChIKey hash codes and the anti-TB effects. These particular algorithms can predict if the drugs have antitubercular effects or not (i.e., binary prediction) by using the InChIKey codes as training data. The current study proposes a machine learning ensemble method prediction of the antitubercular activity of a series of synthesized lupane derivatives, based on drug-drug structural similarity [36] retrieved from each substance’s InChIKey (detailed information is presented in Section 4.2). Table 1 lists the final labels resulting from comparing the average score with the threshold values (i.e., specific for each of the seven algorithms in Section 4.2). Our results indicate that according to the average score (mean value of the total score registered for each compound), out of all assessed compounds, nine derivatives (**4, 5, 13, 14, 16, 17, 18, 20**, and **22**) scored below a 0.5 average, making them, according to the prediction algorithms, weak candidates for a potent tuberculostatic activity. However, these results remain to be validated through in vitro biological assessment studies.

All compounds were also run through a mutual information algorithm, in order to determine a probable tendency of the majority to share common characteristics with one or more compounds that have the same mechanism of tuberculostatic action. This algorithm is a complementary (testing) method, very recently employed in chemistry, which measures the structural similarity between compounds by computing the mutual information (from information theory) between the InChIKey codes. Such approaches were recently used to characterize structure similarity [37] and drug-target compatibility [38]. This phase is necessary in order to narrow the search range for a probable mechanism of action characteristic of the studied lupane derivatives. Table 2 lists the result summary of the mutual information comparison of the tested compounds with the anti-TB and the non-anti-TB drugs from DrugBank 5.1.8 (complete results are available in Table S3). According to the obtained data, 11 out of 20 compounds share high similarity with rifampicin, while 2 other compounds have rifapentine listed as their most similar drug. This accounts for 2 thirds sharing mutual similarity with rifamycins which implies that a possibility that these lupane derivatives share a similar mechanism of action, should be investigated.

### 2.3. MIC Determination against M. Tuberculosis and NTM Strains

The MICs of compounds **1–18**, **20**, and **22** were determined against *M. tuberculosis* H37Rv and three clinically relevant resistant strains using the microbroth dilution method. MIC was determined using optical density and a calorimetric growth indicator. For optical density (OD_600nm_), the MIC is considered the first concentration to inhibit growth when compared to untreated growth control. For the calorimetric growth indicator, the MIC is calculated as the first concentration for the observed color change from pink, indicating active growth to blue, indicating no active growth. In total, 16 compounds demonstrated growth inhibitory activity against *M. tuberculosis* H37Rv in the performed assays (≤32 µM), and 3 compounds were inactive based on no growth inhibition in the ranges tested. Compound **1** was included as reference for the antibacterial activity of the currently assessed compounds, its antimycobacterial effects being previously reported [26,28,29]. Compounds considered very active exhibited MIC values within the micromolar range (up to 10 µM) [39], therefore compounds **2, 7, 9, 10, 16**, and **20** were highly active (≤4 µM). As a result, azepano-lupeol **2** was two-times more active than azepanobetulin **1**, while hydrazido-hydrazone **7** was a lead compound with MIC value 0.5 µM.

Tuberculosis treatment is standardized and updated regularly by the WHO, currently including several drugs generally categorized as first, second, and third line therapeutic options; rifampicin and isoniazid are very active anti-TB drugs included in the first-line category. However, *M. tuberculosis* may acquire spontaneous chromosomal mutations which trigger antibiotic resistance [40]. Once the MTB strain becomes resistant to first-line drugs such as rifampicin and isoniazid, it is catalogued as multidrug resistant and may progress to totally drug-resistant or extremely drug-resistant tuberculosis, when the resistance manifests against the entire first and second-line drugs [41]. In light of these facts, the testing of new drug only on drug-susceptible *M. tuberculosis* may prove insufficient to provide actual treatment choices, in particular for patient with clinical forms of the diseases that have already developed a type of resistance.

Compounds **1–18** and **20**, **22** were tested against three mono-resistant MTB strains: *rpoB*^S450L^, *gyrA*^D94K^ and *katG*^del^, respectively. Strain MTB *rpoB*^S450L^ is characterized by a mutation at codon 450 (S450L) of the beta subunit of DNA-directed RNA polymerase of MTB, rpoB, which is mainly associated with rifampicin resistance [42]. The H37Rv *gyrA*^D94K^ is a *M. tuberculosis* strain resistant to moxifloxacin, which contains a mutation of *gyrA* (D94K) that encodes DNA gyrase subunits and was identified as the most common mechanism to result in fluoroquinolone-resistant tuberculosis [43]. H37Rv *katG*^del^ is an isoniazid mono-resistant strain containing a catalase-peroxidase *(katG)* gene mutant associated with cell wall synthesis [44]. The active compounds demonstrated similar activity against the mono-resistant strains (*rpoB*^S450L^ or *gyrA*^D94K^ or *katG*^del^). All MICs remained the same or within the 4-fold acceptable margin of variation versus mono-resistant H37Rv as tested. Interestingly, some compounds that exerted intermediate or no inhibitory activity against *M. tuberculosis* H37Rv were revealed as stronger inhibitors against the mono-resistant strains. Compounds **6**, **8** exhibited intermediate activity on *M. tuberculosis* H37Rv but were very active on all three mono-resistant strains, compound **3** and **20** exerted intermediate activity on one H37Rv resistant strain (compound **3**, 8 µM on *gyrA*^D94K^, compound **20**, 8 µM *rpoB^S450L^*), while being highly active on the other two. Compounds **13** and **22**, although inactive on *M. tuberculosis* H37Rv, were very or mildly active on all mono-resistant strains, while **14** proved inactive on all strains except for *katG*^del^. Compound **7** was mildly active on the H37Rv *katG*^del^ strain (8 µM) but showed the highest antitubercular activity on all other *M. tuberculosis* tested strains (0.5 µM). Four compounds (**2**, **9**, **10**, and **7)** were highly active against both wild-type susceptible and mono-resistant strains of *M. tuberculosis* H37Rv, and therefore showing potential for future antitubercular drug development. All compounds had MICs considered to be within the therapeutic range, the MICs were greater that the standard antitubercular drugs rifampicin, isoniazid or moxifloxacin. The activity against mono-resistant strains is particularly significant due to the fact that any large bacterial population may naturally contain mutants that are resistant to single drugs. For *M. tuberculosis*, the resistance against rifampicin and isoniazid is developing relatively fast so the extra-cellular population of bacilli will include mono-resistant organisms [45]. All so far corroborated data show that out of the 17 compounds that showed antitubercular activity, 6 were highly active on the H37Rv wild strain, out of which 4 also emerged as moderately active compounds on the three antibiotic resistant strains.

Nontuberculous mycobacteria (NTM) are ubiquitous organisms in the environment, concentrating in particular in soil and water sources, which associate with biofilm formation and increased antibiotic resistance; they are resistant to high temperature and low pH. Among NTM bacteria, the slow growing *M. avium* complex, which encompasses many subspecies and the fast-growing *M. abscessus*, are the most commonly associated with lung disease [46]. The treatment is rather long, depends on the bacterial susceptibility profiles, and lacks evidence-based guidelines, usually consisting in the administration of a combination of at least three drugs; it may lead to severe adverse effects and high probability of relapse [47].

The MICs of compounds **1–18**, **20**, and **22** were determined against the NTM strains *M. avium* (ATCC 25291) and *M. abscessus* (ATCC 19977) using the same microbroth dilution method as mentioned above. In total, 15 compounds demonstrated growth inhibitory activity against *M. abscessus* in the performed assays (≤32 µM) and 4 compounds were inactive based on no growth inhibition in the ranges tested (Table 3). In particular, **2** and **9** were highly active (≤4 µM). Out of the four compounds inactive on *M. abscessuss*, **22**, **15** remained inactive on *M. avium* as well while compounds **14** and **16** revealed strong antitubercular activity; in particular, compound **14** exhibited an MIC value similar to rifampicin (0.0625 µM), thus qualifying as a potential therapeutic alternative. Overall, 17 compounds were proven highly active against *M. avium*, with MIC values ≤4 µM; for compound **1** the MIC value against *M. avium* was identical to clarithromycin (0.125 µM). By comparing the inhibitory activity of the tested compounds with standard antibiotics or chemotherapeutics, one can notice that compounds **2** and **9**, which revealed high activity on both *M. abscessus* and *M. avium* exhibited lower MIC values than clarithromycin or amikacin, respectively, on *M. abscessus.* Collectively, compounds **2** and **9** represent promising therapeutic alternatives to standard drugs as treatment against both NTM strains. Five compounds with MIC values 8 µM may stand as potential antimycobacterial drugs against *M. abscessus* with similar potencies as clarithromycin and amikacin. In terms of *M. avium* infections, most compounds (two exceptions **12**, **15**) might act as therapeutic agents due to their high in vitro activity. However, none of the tested compounds exhibited stronger antimycobacterial effects than the tested standard drugs.

### 2.4. Molecular Docking

**Figure 2:**
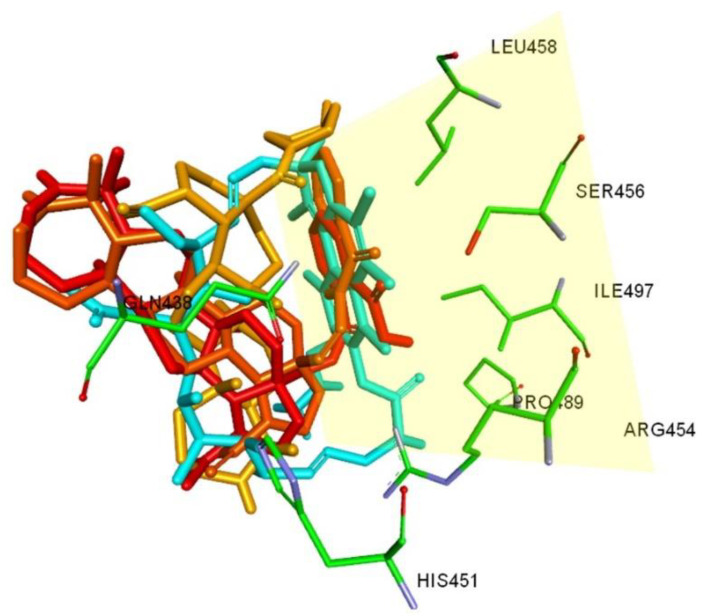
Rifampicin structure (light blue) overlayed on the docked conformations of compounds **4** (red), **7** (dark orange), and **9** (light orange). The highlighted yellow area shows the three compounds with the C28 side chain oriented towards the point of interaction between the rifampicin naphthyl center ring and the surrounding amino acids (green).

Following the resulting data obtained after the mutual information similarity analysis corroborated with the biological antitubercular activity results, we aimed to investigate whether the studied lupane derivatives exhibit rifampicin-like tuberculostatic effect by employing a molecular docking protocol. For this purpose, the lupane type derivatives were docked in the rifampicin target protein, *M. tuberculosis* RNAP. A recent study showed that *M. tuberculosis* RNAP DNA elongation can also be inhibited by targeting a non-overlapping rifampicin binding pocket specific for a class of specific inhibitors called Nα-aroyl-N-aryl-phenylalaninamides (AAPs) [48]. Therefore, the studied compounds were docked in the two adjacent binding pockets using two RNAP conformations, one complexed with rifampicin (PDB ID 5UHB) and the other complexed with an AAP (PDB ID 5UHE). Docking scores show a clear tendency of the lupane derivatives to favor the rifampicin binding site (Table 4). Docking data are in line with the determined MIC values against the H37Rv strain. In both cases compounds **7** and **9** are the top two assessed candidates. Overall, the obtained **∆**G values ranging between −6.3–−8.3 kcal/mol are comparable with the value obtained for the native ligand rifampicin (−10.3 kcal/mol). Most compounds show a similar conformation withing the RNAP binding site. The modified azepano-lupane scaffold is oriented along the space were the rifampicin ansa ring is located, and the C28 side chain is within hydrogen bond forming distance with aminoacid residues like Glu438, Glu435, Arg454 or Ser456 that interact with rifampicin through HB as well (Figure 2). From a structural perspective, an N-containing heterocycle linked to an ester or amide group located in the C28 side chain, proved to beneficially influence the calculated binding energy. This aspect is in most cases also reflected by the in vitro biological activity of the compounds on the *M. tuberculosis* H37Rv strain. According to ligand-protein binding analysis of the tested compounds, the azepane ring N atom does not usually interact by forming HB, being too far from the amino acid residues. Nevertheless, there are some compounds such as **11** and **12**, where the heterocyclic N atom is involved in an amide bond, whose respective oxygen atom acts as HB donor. Structural derivatizations of the isopropenyl group leads to a decrease in the tuberculostatic activity as shown by the MIC values of compounds such as **13**, **14**, **15**, and **16**. This tendency is also reflected on the docking scores of the last three structures. However, this correlation between binding ∆G values and MIC values is not noticeable for all the mentioned compounds due to a limitation of docking methods that can’t take into account other parameters, which might influence the in vitro activity of the discussed substances.

**Figure 3:**
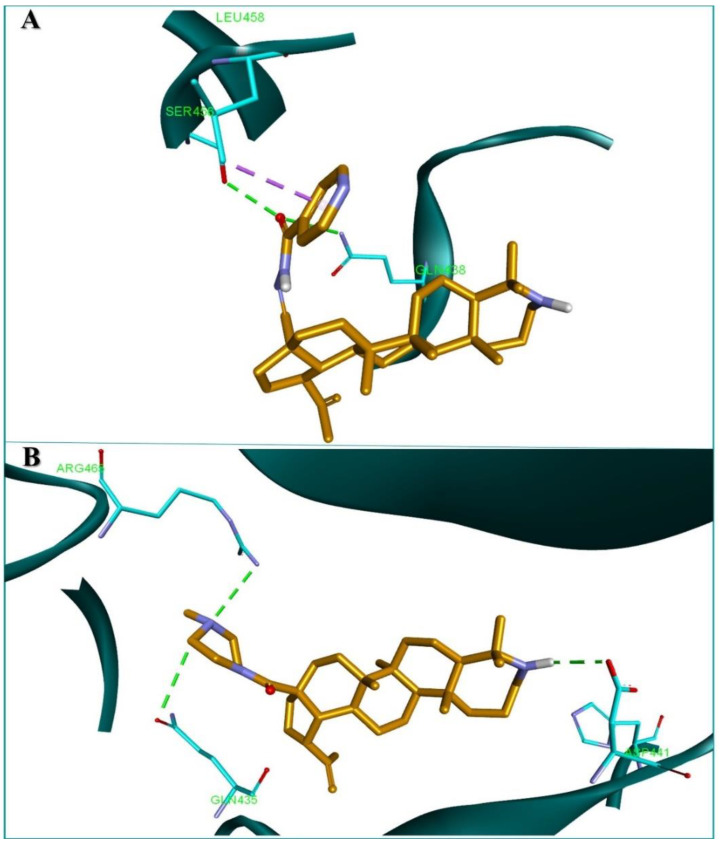
Structure of *M. tuberculosis* RNAP (5UHB) in complex with compound **7** (**A**) and compound **9** (**B**); HB interactions are depicted as green dotted lines and hydrophobic interactions as purple dotted lines; interacting amino acids are shown as light blue sticks and docked compounds are colored in orange.

The two highest active compounds **7** and **9** as indicated by ∆G and MIC (H37Rv) values, interact with the binding site of RNAP mostly through HB formation. Compound **9** is anchored on both opposite sides through 3 HBs with ASP441, Glu435, and Arg465 (Figure 3A) while **7** is one sided bound through its pyridine containing side chain, forming 3 HBs with Ser456 and Glu438 and one hydrophobic interaction with Leu458 (Figure 3B).

Rifampicin resistance is frequently correlated with mutations of the RNAP gene, resulting in various aminoacid substitutions that interfere with the binding affinity of the drug towards its targeted site. Amino acid residue substitutions correlated with rifampicin resistant clinical strains frequently involve His526, Ser531, and Tyr516 [49]. Compounds **7**, **9**, and the rest majority of docked structures do not interact with residues involved in rifampicin resistance-related mutations. This aspect is also reflected in all biologically active compounds that show similar antitubercular activity against both the M. tuberculosis H37Rv (rpoB^S450L^) rifampicin resistant strain and the wild type strain, with the exception of **16** which shows a similar in vitro profile with rifampicin.

Based on all computational evidence correlated with the in vitro antitubercular activity and taking into consideration the obvious limitations of each method, we can preliminarily conclude that the investigated lupane derivatives may exhibit rifampicin-like antitubercular in vitro activity.

### 2.5. Ex Vivo Efficacy and Cytotoxicity

During infection, *M. tuberculosis* is usually phagocytosed by lung macrophages, which provide a suitable environment for its replication. Therefore, any effective antitubercular drug must possess the ability to penetrate host cell [50]. In order to assess if compounds are cell permeable and have intracellular activity, the intracellular activity of compounds was determined ex vivo in THP-1 macrophages against *M. tuberculosis* H37Rv, *M. avium* complex (ATCC 700,891 MAC 101), and *M. abscessus* (ATCC 19977). These standardized assays aimed to assess the growth inhibition of the mycobacteria during drug exposure under ex vivo environment compared to the in vitro determined MICs. The performance of this ex vivo vs. in vitro comparison provides important information regarding the cell permeability of tested drugs as well as their ability to inhibit bacterial growth in an environment that mimics in vivo conditions. The THP-1 line was selected based on several advantages, including whether it can be reliably cultured in vitro, provides a homogenous genetic background (thus reducing results variability), and whether its gene expression can be down-regulated by small interfering RNAs (thereby allowing downstream investigations) [51]. THP-1 cell infected with the mycobacterial strains, respectively, were treated with test compounds at MIC concentrations as determined during in vitro studies. After exposure, the infected THP-1 cells were lysed, and growth inhibition measured compared to an untreated intracellular growth control. Compounds that are considered to have ex vivo activity are able to inhibit >90 ± 5% of the bacterial growth; an intermediate activity is recorded when 50–85% of the growth is inhibited compared to the untreated control. Positive controls isoniazid, rifampin and clarithromycin were included and were within expected ranges with >90% inhibition at their respective MICs.

Eleven of the most in vitro active compounds on the H37Rv strain were tested on THP-1 infected cells. In terms of inhibitory activity against *M. tuberculosis* H37Rv most compounds proved effective with growth inhibition percentages ranging between 60% and 87%; while compound **9** was highly active with a 92% growth inhibition, similar to the ones reported for the antitubercular standard drugs isoniazid and rifampicin (Table 5). The intracellular efficacy of the tested compounds against *M. tuberculosis* infected macrophages indicates a high cell permeability as well as ex vivo efficacy. Previous studies on pentacyclic triterpenoids suggest that the inhibition of intracellular bacteria might depend on a phagocytosis-related mechanism, consisting in the activation of VPS34 complex II by an AMPK-dependent signaling pathway [52]. Future studies are needed in order to fully elucidate the underlying mechanisms for the antitubercular activity.

One can notice that of the eleven compounds tested for ex vivo efficacy against the NTM strains, three of them exhibited a low inhibitory activity (≤64 µM) against *M. avium* and no compound was even mildly active against *M. abscessus*; compound **1** was added as reference. Despite the fact that MTB and NTM share several fundamental cellular and molecular pathophysiological mechanisms, they also differ significantly in terms of host-susceptibility, disease presentation and susceptibility to standard anti-TB drugs, thus requiring a different therapeutic approach [53]. The tested compounds showed strong cell permeability when tested on *M. tuberculosis*-infected THP-1 cells, so we may presume that the same cell permeability was maintained during experiments on NTM-infected THP-1 cells. However, the antimycobacterial activity was not preserved for the most active compounds **9** and **2** against *M. avium*, but remained rather similar for compound **5** and even increased for compound **18**. For compounds **2** and **9** a possible explanation would be that, although cell permeable, the drug did not reach the cell interior in sufficient concentration to effectively kill the NTM mycobacteria. Another potential explanation could be that NTM bacteria possess a cell envelope up to 20 times less permeable than *M. tuberculosis* [54]. The lack of activity against *M. abscessus* was somewhat to be expected since the species shows an intrinsic multidrug resistance [55]. We could therefore conclude that the intracellular activity of the tested compounds does not depend exclusively on their ability to penetrate the mycobacterial wall but also on additional mechanisms that should be further investigated.

Cytotoxicity tests were carried out against three human cell lines, THP-1 (ATCC TIB-202), HepG2 (ATCC HB-8065), and HeLa (ATCC CCL-2) by means of MTT assay. [51]. CC_50_ values were calculated from dose response curves to determine cytotoxicity of test compounds and are reported in Table 6. Using the MIC values obtained through in vitro tests, a selectivity index (SI) was calculated (ratio of CC_50_/MIC) in order to determine toxicity/efficacy relationship. Cytotoxicity testing against THP-1 cells determined that all compounds had CC_50_ values ≤ 16 µM; all compounds had CC_50_ values ≤ 6 µM against HepG2 and CC_50_ values ≤ 18 µM in HeLa cells, respectively (Table 6). Mitomycin C was included as a positive control and values were within expected range. One can notice that in the case of THP-1 cells, most compounds revealed higher values for CC_50_ to the one recorded for standard anticancer drug mitomycin C. However, one exception was revealed: compound **2** was presented higher toxicity in this case. Significant cytotoxic activities as revealed by CC_50_ values within the micromolar range were reported for compound **8** on both HepG2 and HeLa cell lines as well as for compounds **2**, **3**, **9**, and **17** on the HepG2 cell line. The selectivity index was used as a tool to estimate the therapeutic window of the compound; according to several authors, a suitable drug candidate should have a therapeutic index ≥10 in order to exhibit favorable efficacy/toxicity equilibrium [56]. The range of selectivity index values for HepG2 and HeLa were 0.04–3.8 and 0.07–22.5, respectively, while for THP-1 cell line a selectivity index range of 0.15–20.5 was recorded. Overall, we can state that some of the tested compounds show moderate therapeutic selectivity and while compounds with SI below 1 exhibit noticeable toxicity towards human cells and cannot be considered safe for future biological assessment. However, due to the fact that some derivatives revealed selectivity indexes close to 10 or above on certain cell lines (HeLa and THP-1), they may represent the starting point for future semisynthetic derivatives with optimized parameters.

### 2.6. Combinatorial Testing with Standard of Care Drugs against M. tuberculosis H37Rv

One major threat in the treatment of tuberculosis is the emergence of multidrug-resistant mutants, even with the use of drug combinations. One potential solution would be the revival of existing chemotherapeutics via effective combinations with new chemical compounds able to optimize the overall antibacterial activity and prevent the development of resistance. In addition, the combinatorial therapy requires smaller doses of each individual drug thus minimizing adverse effect and overcoming toxicity issues. Synergistic or additive effects between triterpene derivatives and traditional drugs would therefore improve the effectiveness and simultaneously reduce the probability of resistance occurrence for both associated compounds [57,58].

Four compounds were tested in combination with two to three of the standard of care (SoC) drugs rifampin, isoniazid, and ethambutol in order to outline the combinatorial effect of test compounds with SoC drugs when treating *M. tuberculosis* H37Rv in vitro. Compounds were progressed into in vitro fractional inhibitory concentration (FIC) determination against *M. tuberculosis* H37Rv by checkerboard method in microdilution assays. Dual treatments can be synergistic, additive, indifferent, or antagonistic. The results are displayed in Table 7.

FIC testing against *M. tuberculosis* H37Rv demonstrated that compounds had either an additive or indifferent effect when combined with SoC drugs. During previous ex vivo tests, all compounds were revealed with intermediate or high cell permeability. When combined with standard drugs, the triterpene derivatives might enhance cell wall permeability to the traditional drug, thus generating a synergistic effect. The association between tested compounds and standard of care drugs resulting in additive effects can be recommended for compounds with different mechanism of action, bearing the benefit of reducing individual doses and thus adverse effects. The lack of synergistic effects may represent a drawback for the clinical use of the tested combinations. However, no antagonistic effect was recorded in either combination. The extrapolation of in vitro to in vivo results should be further investigated.

### 2.7. Frequency of Resistance

Antimicrobial resistance currently represents a major public health problem around the world and has therefore become a health security concern [59]. Patients with bacterial infections which have become resistant to one or multiple drugs are facing worse clinical outcomes and even deaths. In addition, they may become the source of drug-resistant infections to other people. Therefore, the assessment of the frequency of resistance (FoR) may provide significant information in determining the potential clinical value of a test compound advancing through the drug discovery process. Moreover, the FoR also indicates if resistance development is a rare event and, subsequently, this information may clarify the manner, in which resistance influences the susceptibility-resistance conversion of a strain to other SoC drugs used in a treatment regimen. The approach for selection of resistance employed in the current study affords the FoR to be calculated across different inhibitor concentrations bacterial numbers.

Compounds **1**, **18**, and **22** were used to generate resistant mutants from the drug susceptible strain *M. tuberculosis* H37Rv to calculate the FoR. Since the rate of selection of resistant mutants decisively depends on the concentration of the selecting agent [60], experiments were conducted at 2-, 8-, and 16-times the MIC determined during the in vitro study. Plates were monitored daily for colony formation and when colonies formed, they were enumerated and the FoR was calculated for each drug concentration and bacterial amount by dividing the number of resistant CFU to the total number of CFU inoculated on the respective plate. The results include FoR for each compound submitted at different concentration relative to MIC as appropriate and FoR for each SoC drug, for comparison, at different concentration relative to MIC (Table 8).

Analyzing comparatively the frequencies of resistance of *M. tuberculosis* strain H37Rv against standard control and test sample drug compounds at 2×, 8×, and 16× the MIC, one can notice that the values reported for the lupane derivatives were similar or lower than the ones reported for the standard compounds. At 2× the MIC, the FoR of compounds of compounds **1**, **18** and **22** resembled the one recorded for rifampicin. However, when compared the ethambutole and pretomanid’s (a new drug approved for the treatment of multidrug-resistant TB [61]) FoR, the values registered for compounds **1**, **18** and **22** were 10 times lower, while compared to isoniazid, the FoR was 100 fold reduced (10^−4^ for isoniazid versus 10^−6^ for tested compounds). Bedaquiline is the first drug from a new class, diarylquinolines, approved by FDA for the treatment of multidrug-resistant TB (MDR) [62], with a low FoR; compounds **1**, **18** and **22** revealed similar low FoR values.

At 8× the MIC, the FoR values for the tested compounds were also similar to rifampicin but up to 100 times lower compared to ethambutole, isoniazid, and pretomanid. When 16× the MIC was applied, none of compounds exhibited antibacterial resistance while all standard of care drugs triggered resistance development, in particular isoniazid and pretomanid. This is particularly relevant in terms of clinical applications and outcomes due to the fact that current therapy regimens associate up to four recommended drugs for the main purpose of avoiding bacteria resistance [63] and treatment failure. Thus, the introduction of new drugs with very small potential of bacterial resistance would greatly improve the therapeutic anti-TB arsenal and reduce the number of drugs in recommended associations.

## 3. Conclusions

Our current work reports the biological antimycobacterial investigation of azepano-lupane type derivative series. The obtained results showed that 17 compounds exhibit in vitro antitubercular activity; 6 were highly active on the H37Rv wild type strain, out of which 4 also emerged as highly active compounds on three antibiotic mono-resistant H37Rv strains (*rpoB*^S450L^*, katG*^del^*, gyrA*^D94K^). The highest active structure against the H37Rv strain was compound **7** with a reported MIC of 0.5 µM. Corroborated in silico analysis revealed that the tested active compounds show a rifampicin like antitubercular activity, with compound **7** scoring the highest as a potential *M. tuberculosis* RNAP inhibitor. The series was also tested on NTM strains revealing that most compounds exhibit antimycobacterial activity against *M. avium* with compound **14** recording the same MIC value as the positive control drug rifampicin (0.0625 µM). The tested compounds also revealed ex vivo efficacy on THP-1 MTB infected cells and low cytotoxicity on normal cells, with candidates such as compound **7** showing high selectivity index values making it a good candidate for future antitubercular drug development. Obtained biological data confirm our previous findings that azepano-lupane type triterpenoids represent a promising scaffold for antimicrobial drug research.

## 4. Experimental Part

### 4.1. Chemistry

The spectra were recorded at the Center for the Collective Use “Chemistry” of the Ufa Institute of Chemistry of the UFRC RAS and RCCU “Agidel” of the UFRC RAS. ^1^H and ^13^C-NMR spectra were recorded on a “Bruker *Avance*-III” (Bruker, Billerica, MA, USA, 500 and 125.5 MHz respectively, δ, ppm, Hz) in CDCl_3_, internal standard tetramethylsilane. Mass spectra were obtained on a liquid chromatograph–mass spectrometer LCMS-2010 EV (Shimadzu, Kyoto, Japan). Melting points were detected on a micro table “Rapido PHMK05“ (Nagema, Dresden, Germany). Optical rotations were measured on a polarimeter “Perkin-Elmer 241 MC” (Perkin Elmer, Waltham, MA, USA) in a tube length of 1 dm. Elemental analysis was performed on a Euro EA-3000 CHNS analyzer (Eurovector, Milan, Italy); the main standard is acetanilide. Thin-layer chromatography analyses were performed on Sorbfil plates (Sorbpolimer, Krasnodar, Russian Federation), using the solvent system chloroform-ethyl acetate, 40:1. Substances were detected by 10% H_2_SO_4_ with subsequent heating to 100–120 °C for 2–3 min. Compounds **1** [26], **2** and **3** [29], **4–6, 11** and **12** [30], **7** [32], **8**, **10**, **13** and **18** [33], **9** [34], **14–16** [31], **17** [35], **21** [28] were prepared by the literature methods.

*17-(Propargyl)-3-deoxo-3a-homo-3a-aza-lup-20(29)-en-carboxamide (20).* To a solution of compound 19 [27] (1 mmol; 0.46 g) in CH_2_Cl_2_ (15 mL) (COCl)_2_ (2 mmol; 0.2 mL) was added and stirred at room temperature for 2 h. After reaction completed the solvent was removed *in vacuo*, the crude product was dissolved in CH_2_Cl_2_ (10 mL), propargylamine (1 mmol; 0.06 mL), triethylamine (five drops) were added and a mixture was stirred for 3 h at room temperature. After washing with H_2_O until neutral, the solvent was dried, and evaporated *in vacuo*. The resulting solid was chromatographed over an Al_2_O_3_ column (eluent petroleum ether–chloroform (1:1→0:1)). Beige solid; yield 0.32 g (64%); m.p. 206 °C; [α]*_D_*^20^ +27° (c 0.10, CHCl_3_); ^1^H NMR (500 MHz, CDCl_3_): δ ppm 0.89, 0.91, 0.96, 1.14, 1.23, 1.61 (s, 18H, 6CH_3_), 1.62−3.10 (m, 27H, CH and CH_2_), 2.13 (s, 1H, CH), 3.80−4.05 (m, 2H, CH_2_), 4.50 and 4.65 (both s, 2H, CH_2_), 5.75 (br.s., 1H, NH); ^13^C NMR (125.5 MHz, CDCl_3_): δ 22.3, 22.5, 23.2, 25.6, 26.2, 26.5, 27.3, 28.6, 29.0, 33.1, 36.9, 37.4, 37.9, 40.8, 41.0, 41.9, 42.2, 42.6, 44.0, 46.1, 47.2, 47.4, 47.5, 48.1, 49.6, 54.5, 55.3, 60.0, 70.7 (CH), 80.4 (C), 109.3 (C-29), 150.0 (C-20), 176.1 (C-28) ppm; MS: *m/z* 493 [M+H]^+^. Anal. Calcd for C_33_H_52_N_2_O: C, 80.43; H, 10.64; N, 5.68. Found: C, 80.59; H, 10.54; N, 5.80.

*3-Deoxo-3a-homo-3a-aza-lup-20(29)-en-carboxamide acetate (**22)***. A solution of compound **21** [28] (1 mmol; 0.45 g) in glacial AcOH (5 mL) was refluxed for 3 h, then poured into H_2_O (100 mL), the precipitate was filtered, washed with H_2_O until neutral, and dried. The crude product was chromatographed over an Al_2_O_3_ column (eluent chloroform and chloroform–ethanol, 50:1). Cream solid; yield 0.39 g (79%); m.p. 147 °C; [α]*_D_*^20^ +44° (c 0.05, CHCl_3_); ^1^H NMR (500 MHz, CDCl_3_): δ ppm δ 0.89, 0.95, 1.10, 1.42, 1.58, 1.63 (6s, 18H, CH_3_), 1.20−2.01 (m, 24H, CH and CH_2_), 2.24 (s, 3H, CH_3_), 2.50 (br.s, 1H, H19), 2.91−3.33 (m, 1H), 3.61−3.82 (m, 1H), 4.55 (br.s, 1H, H29a), 4.75 (br.s, 1H, H29b), 5.55 (br.s., 1H, NH); ^13^C NMR (125.5 MHz, CDCl_3_): δ 14.5, 16.4, 16.5, 19.3, 21.2, 21.9, 22.7, 22.8, 23.4, 25.7, 26.9, 27.8, 27.9, 29.6, 30.1, 33.5, 34.9, 37.5, 39.1, 40.6, 41.2, 42.9, 46.7, 47.1, 47.3, 48.7, 54.5, 63.2, 110.0 (C-29), 149.9 (C-20), 170.6 (C-31), 181.91 (C-28); MS: *m/z* 497 [M+H]^+^. Anal. Calc. for C_32_H_52_N_2_O_2_: C, 77.37; H, 10.55; N, 5.64. Found: C, 77.31; H, 10.78; N, 5.75.

### 4.2. Computational Prediction of Tuberculostatic Compound Activity and Mechanism of Action

#### 4.2.1. Machine Learning Based Antitubercular Activity Prediction

**Figure 4:**
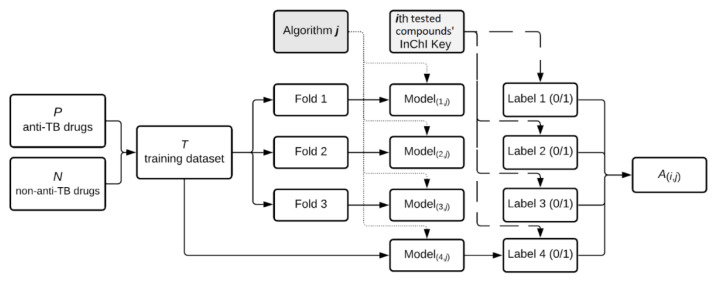
Overview of the machine learning approach: we split the training dataset T (consisting of the reunion of P and N sets) into three subsets, namely Fold 1, Fold 2, and Fold 3. After training a model with an algorithm j with one of the 3 subsets or the entire training set, we obtain a label that we use to compute, for each tested compound i, the final score/label of an algorithm j, the value presented in Table 1 on line i, column j, A(i,j).

In our current study a machine learning ensemble method was employed for the prediction of the antitubercular activity of a series of synthesized lupane derivatives. This process aggregates seven machine learning algorithms, supervised (Linear Regression [64,65], K-Nearest Neighbors [66], Random Forest [67], SVC, Linear SVC, MLP) and unsupervised (K-Means for clustering) [68,69,70]. The validation is a binary classification problem; our goal is to determine whether the lupane type derivatives are antitubercular active or not. To this end, we use a training dataset T with approved drugs extracted from DrugBank 5.1.8 [71]. T is the union of the P and N datasets; the P (positive) dataset contains the anti-TB drugs, and the N (negative) dataset consists of 71 non-anti-TB drugs, randomly selected from each ATC code in DrugBank 5.1.8. (See the lists containing the P and N drugs in Tables S1 and S2). Since there are only a few approved antitubercular drugs, we also apply a K-fold approach for each algorithm, splitting the N dataset into 3 subsets of 24, 24, and 23 drugs. We run each algorithm (for each subset and with the entire T dataset) and compute an aggregate average score of all partial results. Figure 4 presents the workflow, based on an ensemble of machine learning algorithms, we used to compute the binary values in Table 1, specifically matrix A as

(1) $$A\left(i,j\right)=\{\begin{array}{ll} 1, & \mathrm{if}\;\mathrm{algorithm}\;j\;\mathrm{labels}\;\mathrm{the}\;\mathrm{drug}\;i\;\mathrm{as}\;\mathrm{anti}-\mathrm{TB}\\ 0, & \mathrm{if}\;\mathrm{algorithm}\;j\;\mathrm{labels}\;\mathrm{the}\;\mathrm{drug}\;i\;\mathrm{as}\;\mathrm{non}-\mathrm{anti}-\mathrm{TB} \end{array}$$

In the approach presented in Figure 4, the only considered drug features are the 25 letters from the InChIKeys, which we subsequently converted into ASCII integer values. Since the letters A-Z used inside InChIKeys have ASCII values in the 65–90 range, we normalized the integers before applying the ML algorithms to avoid biased features. We used the machine learning Python library Scikit-Learn [69,70] (version 0.24.2) for our ensemble method’s implementation, involving each of the seven algorithms.

#### 4.2.2. Mutual Information-Based Compound-Drug Similarity Prediction

**Figure 5:**
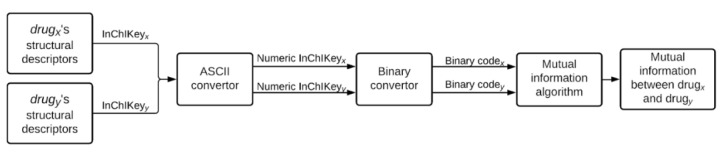
The overview of our method of measuring the structural resemblance between 2 drugs (x and y). For this paper, to confirm the anti-TB properties for some tested compounds, we consider x a test compound and y an approved drug from DrugBank 5.1.8.

Structural resemblance between the lupane type derivatives, on one hand, and both anti-TB and non-anti-TB drugs-on the other hand, was also determined. As such, we use the ASCII (i.e., integer number) expression of the InChIKey molecular strings to represent chemical structure. Then, we measured the amount of structural information shared by two drug codes as the mutual information [72] between the integer strings corresponding to their InChIKey ASCII expressions. By this logic, the tested compounds with higher anti-TB properties should have much larger mutual information with the anti-TB drugs than tested compounds with low anti-TB activity. We present the flow we use to measure the mutual structural information between two drugs (x and y) in Figure 5. We provide all the necessary details for the implementation of our computational confirmation for anti-TB drug properties in tested compounds on the Git repository https://github.com/m1litaru/InChIKeys-project (last commit on 17 September 2021).

#### 4.2.3. Molecular Docking Based Compound Mechanism of Action Assessment

Molecular docking analysis was carried out using a previously described protocol [73,74,75]. Protein structures used as docking targets were retrieved from the RCSB Protein Data Bank [76] (Table 9). Target structures were optimized as docking suitable file formats, using Autodock Tools v1.5.6 (The Scripps Research Institute, La Jolla, CA, USA). All water molecules, unnecessary protein chains and the co-crystalized ligands were removed from the protein structure file, after which Gesteiger charges were added. The target was saved as the required .pdbqt file format, when the software automatically adds polar hydrogen atoms to the structure or merges nonpolar hydrogens, where applicable. Analyzed ligand molecules corresponding to the 19 lupane type derivatives were sketched as 2D structures, using Biovia Draw (Dassault Systems Biovia, San Diego, CA, USA) and were after converted into 3D structures using PyRx’s embedded Open Babel function, using the ghemical force field for compound geometry optimization. Molecular docking was performed with PyRx v0.8 (The Scripps Research Institute, La Jolla, CA, USA), using Autodock Vina’s embedded scoring function [77]. Docking method validation was achieved by re-docking the native ligands into their original protein binding sites. The predicted docking pose was compared with the experimental binding pose. Docking studies were performed for each case only if the root mean square deviation (RMSD) values between the native ligand’s experimental and docked pose did not exceed a 2 Å threshold. The search space grid box was defined in terms of coordinates and size (Table 9) to best fit the binding site. Obtained data for docked compounds was recorded as free binding energy values (ΔG, kcal/mol). Ligand-protein binding interactions were analyzed using Accelerys Discovery Studio 4.1 (Dassault Systems Biovia, San Diego, CA, USA).

### 4.3. Compound Sample Preparation for In Vitro Biological Assessment

Compound stock solutions were prepared at a starting concentration of 1.28 mg/mL in DMSO for tested compounds against *M. tuberculosis* and 2.56 µM in DMSO for tested compounds against NTMs. Aliquots of these stock solutions were prepared a day before in order to ensure dissolution, and stored at −20 °C for single use. Compound dilution master plates (MDP) are then prepared separately as two-fold serial dilutions in Nunc brand polypropylene test plates (target range is adjusted based on the anticipated activity of the molecule under test or for TB from 32–0.12 μg/mL or for NTM from 64–0.06 μg/mL). From the MDP, 2.5 μL portions were transferred to the corresponding wells of MIC test plate containing 0.1 mL bacterial suspension, thus resulting in a final 40× dilution factor. One column on the microtiter plate contains only diluent and no compound and thus serves as a growth control.

### 4.4. MIC Determination against M. tuberculosis Strains

The antimicrobial activity MICs of the tested compounds against M. *tuberculosis* strains was determined by broth microdilution assay using 7H9 Mycobacteria ADC Enriched Medium (Becton Dickinson, 271,310, supplemented with albumin, dextrose, and catalase). Firstly, compounds were prepared in a 96-well MDP following a two-fold serial dilution protocol, starting with column 2 (designated to be the start of each dilution series), where 100 μL of test compounds were added. 100% DMSO was used as the intermediate diluent (50 μL) in each well starting with column 3. The wells of column 11 were only filled with DMSO (growth control). Secondly, in order to measure the MICs of the tested compounds, 2.5 μL of each dilution previously obtained in the MDP were transferred to the corresponding wells in a final MIC assay 96-well plates containing 100 μL 7H9 Mycobacteria ADC Enriched Medium. To ensure minimal evaporation of the medium during incubation, the outer perimeter wells were filled with sterile water. The plates were inoculated with 5 × 10^5^ CFU/mL *M. tuberculosis* and incubated at 37 °C, 5% CO_2_. The bacterial growth was measured by OD_600_ on day 7/8 using a BioTek™ SynergyH4 plate reader. Subsequently, to assess cell viability, 10 μL AlamarBlue dye were added to each analytical well and the plates were incubated for 24 h. All assay plates were scanned on a flatbed color scanner to detect the changes of well color, from blue (AlamarBlue oxidized form), suggesting no bacterial growth, to pink (AlamarBlue reduced form) that indicates bacterial growth. The lowest concentrations of compounds that blocked the color change from blue to pink were established as the MICs.

### 4.5. MIC Determination against NTM Strains

Compound solutions were prepared at a starting concentration of 2.56 mg/mL in DMSO. In the first column of a 96-well MDP, 200 μL of the compounds were added and serially diluted 1:2 by transferring 100 μL from well to well until column 10, establishing a test range of 64–0.06 μg/mL. The wells of column 11 were filled only with DMSO (growth control). The measurement of MICs of the tested compounds against NTM strains was performed by using the same microbroth dilution method as described above, in terms of MIC assay plate preparation, bacterial growth measurement, and MIC determination. Each assay plate contained negative control wells (bacteria only and media only), positive drug control wells (clarithromycin-CLA and amikacin-AMI), as well as an optional *E. coli* control well.

### 4.6. Ex Vivo Testing

#### 4.6.1. In Vitro Infection of Differentiated THP-1 Human Macrophages

Human acute monocytic leukemia cell lines THP-1 cell lines were seeded in 96-well plates (1 × 10^5^/200 μL medium/well) with complete growth medium RMPI -1640 (ATCC 30-2001) supplemented with 100 nM phorbol 12-myristate 13-acetate (PMA). The plates were then incubated at 37 °C with 5% CO_2_ for 72 h during which on day two, the PMA-enriched medium was replaced with fresh complete medium without PMA. To ensure a mid-logarithmic growth phase of the mycobacteria cell cultures, OD_600_ measurements were correlated with CFU/mL quantification. Thus, the OD_600_ of *M. tuberculosis* H37Rv, *M*. *avium* 101 and *M*. *abscessus* 19,977 was checked. Based on the results, for *M*. *tuberculosis* H37Rv an OD_600_ of 1 was equivalent to ~ 3 × 10^8^ CFU/mL and an OD_600_ of 0.4 to ~2 × 10^7^ CFU/mL. For *M*. *avium* 101 an OD_600_ of 0.3 was equivalent to ~ 1 × 10^9^ CFU/mL and for *M*. *abscessus* 19,977 an OD_600_ of 0.05 was equivalent to ~ 4.9 × 10^6^ CFU/mL. The inoculum was prepared afterwards in complete growth medium at a concentration of 5 × 10^6^ CFU/mL, in order to obtain an infection medium with a multiplicity of infection (MOI) of 10. THP-1 cells were washed with PBS, infected with 0.2 mL infection medium/well and incubated at 37 °C with 5% CO_2_ for 4 h. The cells were then washed twice with 0.2 mL PBS and the medium replaced with a fresh complete growth medium with the tested compounds. The plates were sealed and incubated at 37 °C with 5% CO_2_ for 24 h. The efficacy of the tested compounds was quantified using the colorimetric indicator resazurin (Sigma R7017). Following two washing steps with PBS, the medium was replaced with 7H9 medium; 20 μL of resazurin working solution (0.8 mg/mL mixed with sterile water and Tween-80 in a 2:1:1 ratio) was added to the culture wells. The absorbance was recorded at both 570 nm and 600 nm (BioTek™ SynergyH4 plate reader) for *M. tuberculosis* H37Rv strain after seven days incubation and for NTM strains after three to five days of incubation. The percent growth reduction was calculated using the following formula:

$$\frac{\left(\varepsilon \mathrm{OX}\right)\lambda_{2}{A\lambda }_{1}-\left(\varepsilon \mathrm{OX}\right)\lambda_{2}{A\lambda }_{1}\mathrm{of}\;\mathrm{test}\;\mathrm{agent}\;\mathrm{dilution}}{\left(\varepsilon \mathrm{OX}\right)\lambda_{2}{A\lambda }_{1}-\left(\varepsilon \mathrm{OX}\right)\lambda_{2}{A\lambda }_{1}\mathrm{of}\;\mathrm{untreated}\;\mathrm{positive}\;\mathrm{growth}\;\mathrm{control}}\times 100$$

where:

ε_OX_= molar extinction coefficient of resazurin oxidized form

A= absorbance of test wells

A∘= absorbance of positive growth control wells

λ_1_= 570 nm

λ_2_= 600 nm

#### 4.6.2. Cytotoxicity Evaluation

Cytotoxicity was assessed by using the colorimetric MTT (Methylethiazole tetrazolium-Sigma M6494) and resazurin (Sigma R7017) assay. THP-1, HepG2 and HeLa cells (1 × 10^5^ cells/mL) were cultured in 96-well plates and incubated at 37 °C with 5% CO_2_ overnight. The tested compounds were prepared in a separate 96-well plate as two-fold serial dilutions (tested range: 128–0.0625 μM) and incubated in the same conditions. A volume of 200 μL was transferred from the compound plate to the corresponding wells of the tested cell plate and again incubated for 24 h. Each microtiter plate included blank wells (media only), control wells (cells + media) and positive control wells (mitomycin C treated cells-Sigma M0503). Following the incubation period, the media with compounds was replaced with 100 μL/well of fresh medium (without compounds). Half of the wells of the test plate were treated with 10 µL/well MTT (12 mM), followed by the addition of 100 µL/well detergent solution (0.1g/mL SDS in 0.01M HCl), and half with 10 µL/well resazurin. After 4 h of incubation, the OD_570_ was measured using a microplate reader (BioTek™ SynergyH4 plate reader). For statistical significance, all experiments were performed in triplicate. The percentage cytotoxicity was calculated by using the following formula:

$$\%\mathrm{Cytotoxicity}=\frac{\mathrm{corrected}\;\mathrm{control}\;\mathrm{OD}-\mathrm{correted}\;\mathrm{sample}\;\mathrm{OD}}{\mathrm{corrected}\;\mathrm{control}\;\mathrm{OD}}$$

CC_50_ values were obtained by using a nonlinear regression analysis of the obtained data (GraphPad Prism 6, San Diego, CA, USA).

### 4.7. Antimicrobial Synergy Study against M. tuberculosis H37Rv

The in vitro synergic effect of compounds with two to three of the standard of care (SoC) drugs rifampin, isoniazid, and ethambutol was obtained by using the checkboard dilution test. In brief, for each compound combination, two 96-well compound plates (25 μL/well) were prepared in parallel, following a 2-fold serial dilution across the plates using 7H9 Mycobacteria ADC Enriched Medium and starting at 4× the final desired concentration. For compound 1, from the combination the dilution followed a horizontal pattern from left to right (columns 2–10) whilst compound 2 was diluted from top to bottom (rows B-G). Column 11 and row G were filled only with media and considered control wells. All content from the two plates were transferred to the corresponding wells in a final assay plate (50 μL/well). *M. tuberculosis* H37Rv culture (5 × 10^5^ CFU/mL) was added at equal volume (total volume 100 μL/well). The plates were sealed and incubated at 37 °C for 7/8 days, after which OD_600_ was measured (BioTek™ SynergyH4 microplate reader). The susceptibility of drug combinations against *M. tuberculosis* H37Rv strains was determined by means of Alamar Blue assay, as described in detail above (see MIC determination against *M. tuberculosis* strains). More precisely, based on MIC values, the impact on potency of tested compounds/standard anti-TB association was compared to their individual activities by using the fractional inhibitory concentration index value (FIC), calculated according to the following formula:

$$\mathrm{FIC}\;\mathrm{index}={\mathrm{FIC}}_{a}+{\mathrm{FIC}}_{b}=\frac{{\mathrm{MIC}}_{\mathrm{ab}}}{{\mathrm{MIC}}_{a}}+\frac{{\mathrm{MIC}}_{\mathrm{ab}}}{{\mathrm{MIC}}_{b}}$$

where:

FIC_a_ = Fractional Inhibitory Concentration index value of each compound individually

FIC_b_= Fractional Inhibitory Concentration index value of standard anti-TB individually

MIC_ab_= MIC of each tested compound in combination with standard anti-TB

MIC_a_= MIC of each compound individually

MIC_b_ = MIC of each standard anti-TB individually

FIC index values were interpreted according to a conventional model suggested by EUCAST (2000), as follows:

FIC ≤0.5 = synergy; FIC >0.5- = additive; FIC >1 to <2 = indifference and FIC ≥2 = antagonism; isobolograms were created to show inhibitory relationship between compounds over the dilution series [78].

### 4.8. Assessment of Frequency of Resistance

*M. tuberculosis* H37Rv cultures were diluted 1:100 in fresh Middlebrook 7H9 liquid broth medium (Becton Dickinson, 271310) and grown to an OD_600_ of ~1.0 (~1 × 10^8^ CFU/mL). Aliquots of 200 μL inoculum, adjusted to an OD_600_ of 0.25, 0.50, and 1.00, respectively, were then plated onto Middlebrook 7H10 solid agar medium. Compounds were added at two-, eight-, and sixteen-times the MIC value and the plates were incubated at 37 °C and 150 rpm for three-to-four weeks. Separate plates with ten-fold serial dilutions of 0.25, 0.50, and 1.00 inoculums, in compound-free 7H10 solid agar medium, served as control plates. Following the incubation period, each drug resistant colony was transferred to a 4.00 mL aliquot with fresh 7H9 liquid broth media and the equivalent concentrations of drug compounds used in the initial selection (on 7H10 solid agar medium). To confirm resistance, the plates were incubated for another two weeks at 37 °C and 150 rpm. FoR was calculated at 2-, 8-, and 16-times the MIC of each compound concentration by dividing the number of compound resistant colonies (confirmed by subsequent growth in Middlebrook 7H9 liquid broth) by the corresponding CFU plated.

## Figures and Tables

**Table 1:** The predicted label for each of tested compound (i.e., 0 means lack of anti-TB activity, and 1 means anti-TB activity), using K-Means, Linear Regression, K-Nearest Neighbors, Random Forest, SVC, Linear SVC, and MLP machine learning algorithms. The last column presents the average label value, representing the aggregated score. Compounds with a 0.5 lower aggregated score are highlighted in red.

Compound	KMeans	Linear Regression	KNeigh	MLP	SVC	Linear SVC	Random Forest	Aggregated Score
**1**	1	1	0	0	1	1	0	0.57
**2**	1	0	1	0	1	0	1	0.57
**3**	1	0	1	0	1	1	0	0.57
**4**	0	0	0	0	0	1	0	0.14
**5**	0	0	0	0	0	1	0	0.14
**6**	1	0	0	1	1	1	1	0.71
**7**	0	1	1	1	1	1	1	0.86
**8**	0	0	1	1	1	0	1	0.57
**9**	0	0	1	1	1	1	1	0.71
**10**	0	1	1	1	1	1	0	0.71
**11**	0	1	1	1	1	1	1	0.86
**12**	1	0	1	1	0	1	1	0.71
**13**	1	0	0	0	0	0	0	0.14
**14**	0	0	1	1	0	0	0	0.29
**15**	1	0	0	1	1	1	0	0.57
**16**	1	0	0	0	0	0	0	0.14
**1** **7**	0	0	1	0	0	1	0	0.29
**1** **8**	0	0	1	1	0	0	0	0.29
**20**	0	0	0	0	0	0	1	0.14
**22**	0	0	0	0	0	0	1	0.14

**Table 2:** The average score (after normalization) of mutual information values between each tested compound and the anti-TB drugs. The last three columns present the most similar approved anti-TB drugs with the subjected compounds (i.e., the approved anti-TB drug having the highest mutual information with an assessed compound). Compounds in the “Most similar drug” column are highlighted based on their chemical class.

Compound	Most Similar Drug	Second Most Similar Drug	Third Most Similar Drug
**1**	Pyrazinamide	Rifampicin	Protionamide
**2**	Cycloserine	Protionamide	Rifapentine
**3**	Rifampicin	Protionamide	Rifapentine
**4**	Rifapentine	Protionamide	Protionamide
**5**	Rifampicin	Protionamide	Ethambutol
**6**	Rifampicin	Pyrazinamide	Rifapentine
**7**	Protionamide	Terizidone	Enviomycin
**8**	Terizidone	Rifampicin	Protionamide
**9**	Rifampicin	Protionamide	Rifapentine
**10**	Rifampicin	Pyrazinamide	Enviomycin
**11**	Rifapentine	Protionamide	Protionamide
**12**	Rifampicin	Protionamide	Rifapentine
**13**	Protionamide	Pyrazinamide	Enviomycin
**14**	Rifampicin	Protionamide	Rifapentine
**15**	Rifampicin	Terizidone	Streptomycin
**16**	Rifampicin	Protionamide	Pyrazinamide
**17**	Rifampicin	Protionamide	Rifapentine
**18**	Rifampicin	Rifapentine	Streptomycin
**20**	Protionamide	Terizidone	Rifampicin
**22**	Protionamide	Rifamycin	Rifampicin

**Table 3:** Minimal inhibitory concentration of compounds **1–18**, **20**, and **22** against *M. tuberculosis,* mono-resistant MTB and NTM strain panels; active compounds are highlighted from low active (light yellow) to highly active (red).

Compound	MIC (µM)					
	H37Rv	H37Rv (*rpoB*^S450L^)	H37Rv (*katG*^del^)	H37Rv (*gyrA*^D94K^)	*M. abscessus* 19977	*M. avium* 25291
**1**	4 *	4 *	2 *	4 *	8 *	0.125 *
**2**	4	4	4	4	4	1
**3**	8	4	4	8	16	2
**4**	16	8	8	16	16	4
**5**	8	8	4	8	16	1
**6**	8	4	4	4	8	1
**7**	0.5	0.5	8	0.5	8	4
**8**	8	4	4	4	32	4
**9**	2	2	1	2	2	4
**10**	4	4	4	4	8	2
**11**	8	4	8	8	16	4
**12**	32	32	32	>32	8	4
**13**	32	8	8	16	16	2
**14**	>32	>32	16	>32	>64	0.0625
**15**	>32	>32	32	>32	>64	64
**16**	4	32	2	2	>64	4
**17**	16	8	8	16	16	0.5
**18**	16	16	16	8	32	4
**22**	>32	4	16	>32	>64	64
**20**	4	8	4	4	16	4
Rifampin	≤0.004	>1	≤0.004	≤0.004	ND	0.0625
Isoniazid	0.03	0.06	>2	0.06	-	-
Moxifloxacin	0.06	0.03	0.03	>2	-	-
Pretomanid	0.125	0.06	0.06	0.125	-	-
Clarithromycin	-	-	-	-	4	0.125
Amikacin	-	-	-	-	8	ND

* data reported in [26,27,28].

**Table 4:** Recorded docking scores (binding energy, ∆G kcal/mol) for compounds **1–18**, **20**, and **22**. The top 2 ranked active compounds are highlighted.

	Protein PBD ID	5UHB	5UHE
Ligand		Binding Free Energy ∆G (kcal/mol)	
Native ligand		−10.3	−10.9
Compound **1**		−7	−8
Compound **2**		−7.1	−6.5
Compound **3**		−6.3	−5.6
Compound **4**		−7.9	−6.0
Compound **5**		−7.4	−5.6
Compound **6**		−7.6	−6.1
Compound **7**		−8.3	−2.5
Compound **8**		−7.2	−7.6
Compound **9**		−8.2	−4.5
Compound **10**		−7.4	−5.6
Compound **11**		−8	−4.7
Compound **12**		−7.2	−4.3
Compound **13**		−7.8	−9.0
Compound **14**		−6.9	−4.5
Compound **15**		−7.2	−4.8
Compound **16**		−6.9	−4.8
Compound **17**		−6.7	−4,8
Compound **18**		−6.9	−5.1
Compound **20**		−7.6	−5.9
Compound **22**		−7.8	−3.5

**Table 5:** Ex vivo results for the tested compounds. The compounds.

Compound	*M. avium* Ex Vivo Efficacy (>90% Inhibition µM)	*M. abscessus* Ex Vivo Efficacy (>90% Inhibition µM)	*M. tuberculosis* Ex Vivo Efficacy (% Inhibition at MIC)
**1**	8	>128	ND
**2**	>128	>128	87%
**3**	>128	>128	68%
**5**	32	>128	84%
**7**	128	>128	86%
**8**	16	>128	60%
**9**	>128	>128	92%
**10**	64	>128	86%
**17**	>128	>128	87%
**18**	128	>128	80%
**22**	128	>128	ND
Clarithromycin	8	128	ND
Isoniazid	ND	ND	91%
Rifampin	ND	ND	93%

**Table 6:** Cytotoxicity results and calculated SI for the tested compounds on THP-1, HepG2, and HeLa cell lines.

Compound	THP-1 Cytotoxicity (CC_50_ µM)	THP-1 SI	HepG2 Cytotoxicity (CC_50_ µM)	HepG2 SI	HeLa Cytotoxicity (CC_50_ µM)	HeLa SI
**2**	2.006	0.5	0.3305	0.08	2.064	0.52
**3**	8.046	1	0.5936	0.07	2.767	0.34
**5**	15.78	1.97	5.914	0.74	17.73	2.22
**7**	10.25	20.5	1.917	3.83	11.26	22.52
**8**	4.39	0.55	0.7154	0.09	0.9302	0.46
**9**	3.334	1.66	0.2422	0.12	2.419	1.21
**10**	11.54	2.88	1.522	0.38	9.542	2.38
**17**	7.534	0.47	0.7124	0.04	3.771	0.24
**18**	13.03	0.81	1.098	0.07	8.893	0.55
**22**	4.972	0.15	1.432	0.09	2.21	0.07
Mitomycin C	3.094		16.6		8.89	

**Table 7:** FIC Values *M. tuberculosis* H37Rv for compounds **3**, **7**, **9** and **22**.

Compound	Rifampin	Isoniazid	Ethambutol
**3**	1.0 Additive	1.0 Additive	1.5 Indifferent
**7**	1.0 Additive	0.8 Additive	1.0 Additive
**9**	1.5 Indifferent	N/A	2.5 Indifferent
**22**	1.0 Additive	N/A	1.5 Indifferent

N/A–Compounds not approved for testing with this SoC; FIC VALUE ≤ 0.5 (Synergistic); 0.5–1.0 (Additive); 1.0–4.0 (Indifferent); > 4.0 (Antagonistic).

**Table 8:** Frequencies of resistance of *M. tuberculosis* strain H37Rv against standard control and test sample drug compounds at 2×, 8×, & 16× the MIC on 7H10 agar; i.e., µM for compounds **1**, **18** and **22**.

Compound	~10^5^ CFU Plated	~10^6^ CFU Plated	~10^7^ CFU Plated
2× the MIC = 4.00 µg mL^−1^ Bedaquiline (BDQ)	0	3.13 ×10^−7^	1.43 ×10^−7^
8× the MIC = 16.00 µg mL^−1^ Bedaquiline (BDQ)	0	3.13 ×10^−7^	3.57 ×10^−8^
16× the MIC = 32.00 µg mL^−1^ Bedaquiline (BDQ)	0	0	3.57 ×10^−8^
2× the MIC = 2.00 µg mL^−1^ Ethambutol (EMB)	9.44 ×10^−5^	2.78 ×10^−5^	3.61 ×10^−6^
8× the MIC = 8.00 µg mL^−1^ Ethambutol (EMB)	1.11 ×10^−5^	3.75 ×10^−6^	8.57 ×10^−7^
16× the MIC = 16.00 µg mL^−1^ Ethambutol (EMB)	0	0	1.79 ×10^−7^
2× the MIC = 0.25 µg mL^−1^ Isoniazid (INH)	1.39 ×10^−4^	3.75 ×10^−5^	5.00 ×10^−6^
8× the MIC = 1.00 µg mL^−1^ Isoniazid (INH)	5.56 ×10^−5^	3.31 ×10^−5^	3.93 ×10^−6^
16× the MIC = 2.00 µg mL^−1^ Isoniazid (INH)	1.67 ×10^−5^	2.63 ×10^−5^	2.71 ×10^−6^
2× the MIC = 2.00 µg mL^−1^ Ofloxacin (OFX)	0	1.56 ×10^−6^	5.36 ×10^−7^
8× the MIC = 8.00 µg mL^−1^ Ofloxacin (OFX)	0	9.38 ×10^−7^	7.14 ×10^−8^
16× the MIC = 16.00 µg mL^−1^ Ofloxacin (OFX)	0	0	3.57 ×10^−8^
2× the MIC = 1.00 µg mL^−1^ Pretomanid (PRT)	6.94 ×10^−5^	1.06 ×10^−5^	1.79 ×10^−6^
8× the MIC = 4.00 µg mL^−1^ Pretomanid (PRT)	3.89 ×10^−5^	6.88 ×10^−6^	8.57 ×10^−7^
16× the MIC = 8.00 µg mL^−1^ Pretomanid (PRT)	8.33 ×10^−6^	3.13 ×10^−7^	1.43 ×10^−7^
2× the MIC = 1.00 µg mL^−1^ Rifampicin (RIF)	2.78 ×10^−6^	3.13 ×10^−6^	5.36 ×10^−7^
8× the MIC = 4.00 µg mL^−1^ Rifampicin (RIF)	0	3.13 ×10^−7^	7.14 ×10^−8^
16× the MIC = 8.00 µg mL^−1^ Rifampicin (RIF)	0	0	3.57 ×10^−8^
2× the MIC = 8.00 µg mL^−1^ compound 1	2.78 ×10^−6^	3.13 ×10^−7^	1.07 ×10^−7^
8× the MIC = 32.00 µg mL^−1^ compound 1	0	9.38 ×10^−7^	7.14 ×10^−8^
16× the MIC = 64.00 µg mL^−1^ compound 1	0	0	0
2× the MIC = 32.00 µg mL^−1^ compound 18	5.56 ×10^−6^	7.19 ×10^−6^	4.64 ×10^−7^
8× the MIC = 128.00 µg mL^−1^ compound 18	0	3.13 ×10^−7^	3.57 ×10^−8^
16× the MIC = 256.00 µg mL^−1^ compound 18	0	0	0
2× the MIC = 64.00 µg mL^−1^ compound 22	2.78 ×10^−6^	1.25 ×10^−6^	5.36 ×10^−7^
8× the MIC = 256.00 µg mL^−1^ compound 22	0	1.56 ×10^−6^	1.07 ×10^−7^
16× the MIC = 512.00 µg mL^−1^ compound 22	0	0	0

**Table 9:** Molecular docking parameters and protein targets.

Protein	PDB ID	Grid Box Center Coordinates	Grid Box Size	Conformers Generated Per Ligand
Mycobacterium tuberculosis RNA polymerase (Mtb RNAP)-rifampin complex	5UHB	Center_x = 164.393813216 center_y = 162.642568841 center_z = 18.7402222979	size_x = 17.2315566064 size_y = 25.0 size_z = 19.8647468819	8
Mycobacterium tuberculosisRNA polymerase- N-aroyl-N-aryl-phenylalaninamides (AAPs) complex	5UHE	Center_x = −33.0902019309 center_y = −21.9465676304 center_z = 13.5807318	size_x = 14.3058881805 size_y = 25.0 size_z = 13.9275973862	8

## Supplementary Materials

The following are available online at https://www.mdpi.com/article/10.3390/ijms222212542/s1. Figures S1–S4: NMR spectra; Tables S1–S3: Training data.
