# Supplementary material for: New Investigations with Lupane Type A-Ring Azepane Triterpenoids for Antimycobacterial Drug Candidate Design
Source: Int J Mol Sci. 2021 Nov 21;22(22):12542. doi: 10.3390/ijms222212542 (PMC8621456; doi:10.3390/ijms222212542)
Supplement: Supplementary file 1 [file ijms-22-12542-s001.zip › ijms-1426370-supplementary.pdf]

Supplementary Material

New investigations with lupane type A-ring azepane triterpenoids for antimycobacterial drug candidate design

Oxana Kazakova[1*#], Roxana Racoviceanu[2,3#], Anastasiya Petrova[1], Marius Mioc[2,3], Adrian Militaru[4], Lucreția Udrescu[5], Mihai Udrescu[4], Adrian Voicu[6*], Jason Cummings[7], Gregory Robertson[7], Diane Ordway[7], Richard Slayden[7], Codruța Soica[2,3]

[1]Ufa Institute of Chemistry of the Ufa Federal Research Centre of the Russian Academy of Sciences, pr. Oktyabrya 71, 450054 Ufa, Russian Federation
[2] Department II-Pharmaceutical Chemistry, Faculty of Pharmacy, "Victor Babeş" University of Medicine and Pharmacy Timişoara, 2 Eftimie Murgu Sq., 300041 Timişoara, Romania
[3]Res Ctr Pharmacotoxicol Evaluat, Fac Pharm, Victor Babes Univ Med & Pharm Timisoara, Eftimie Murgu Sq 2, RO-300041 Timisoara, Romania
[4]Department of Computer and Information Technology, University Politehnica of Timişoara, 2 Vasile Pârvan Blvd., 300223 Timişoara, Romania
[5]Department I-Drug Analysis, Faculty of Pharmacy, "Victor Babeş" University of Medicine and Pharmacy Timişoara, 2 Eftimie Murgu Sq., 300041 Timişoara, Romania
[6]Department III-Informatics and Medical Biostatistics, Faculty of Pharmacy, "Victor Babeş" University of Medicine and Pharmacy Timişoara, 2 Eftimie Murgu Sq., 300041 Timişoara, Romania
[7]Department of Microbiology, Immunology & Pathology, Colorado State University, 1619 Campus Delivery, Fort Collins, CO 80523-1601, United States

[#] Authors with equal contribution jointly sharing the first author position
* Corresponding authors

Table of content

Figures S1 and S2. Compound (**20**) ^{1}H and ^{13}C NMR spectra ($CDCl_3$) 3

Figures S3 and S4. Compound (**22**) ^{1}H and ^{13}C NMR spectra ($CDCl_3$) 4

Table S1. Training data set for antiTB activity prediction – antiTB drugs 5
Table S2. Training data set for antiTB activity prediction – non-antiTB drugs 5

Table S3. Lupane derivatives – antiTB drug mutual information complete results 9

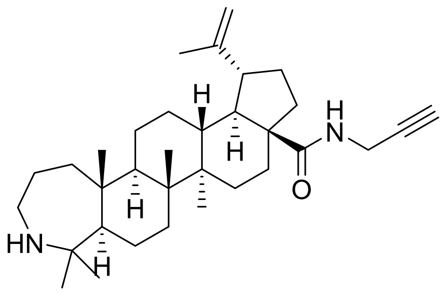

17-(Propargyl)-3-deoxo-3a-homo-3a-aza-lup-20(29)-en-carboxamide (**20**)

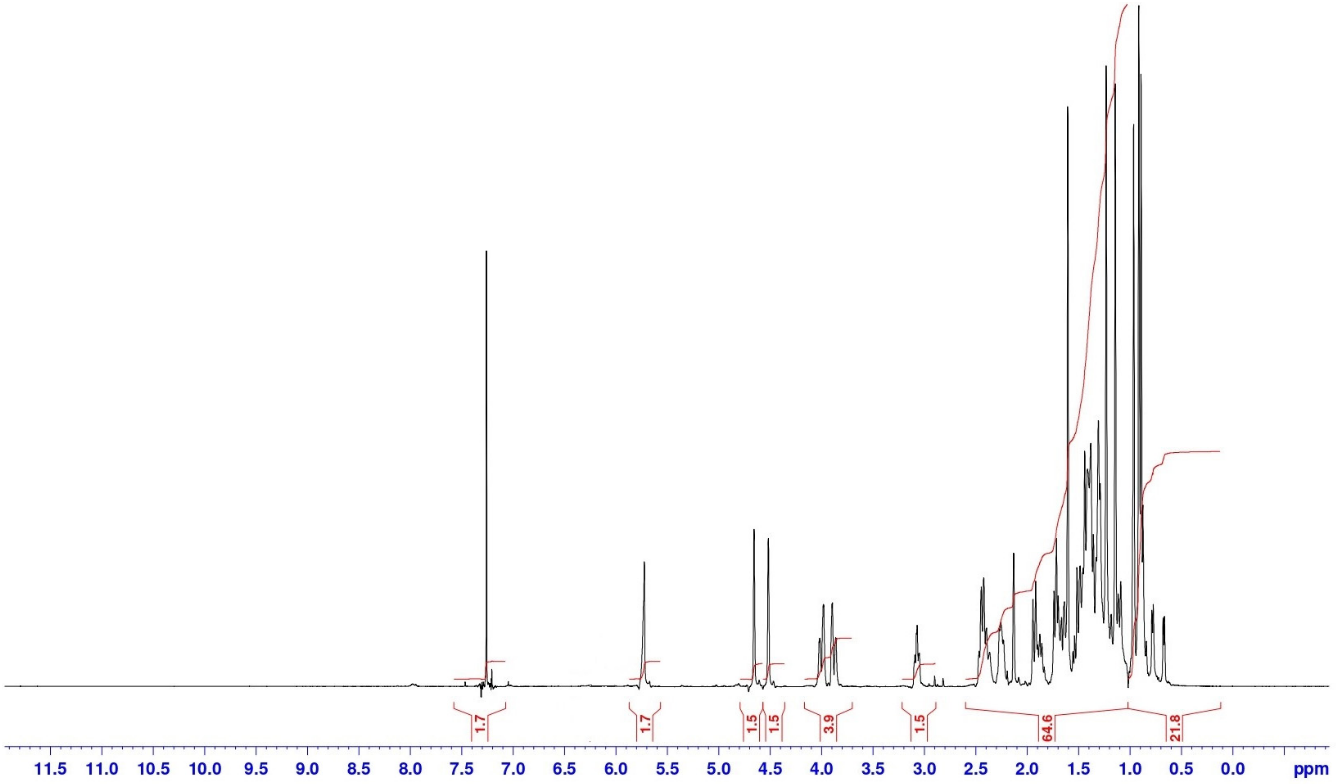

Figure S1. ^{1}H NMR spectrum of compound **20**

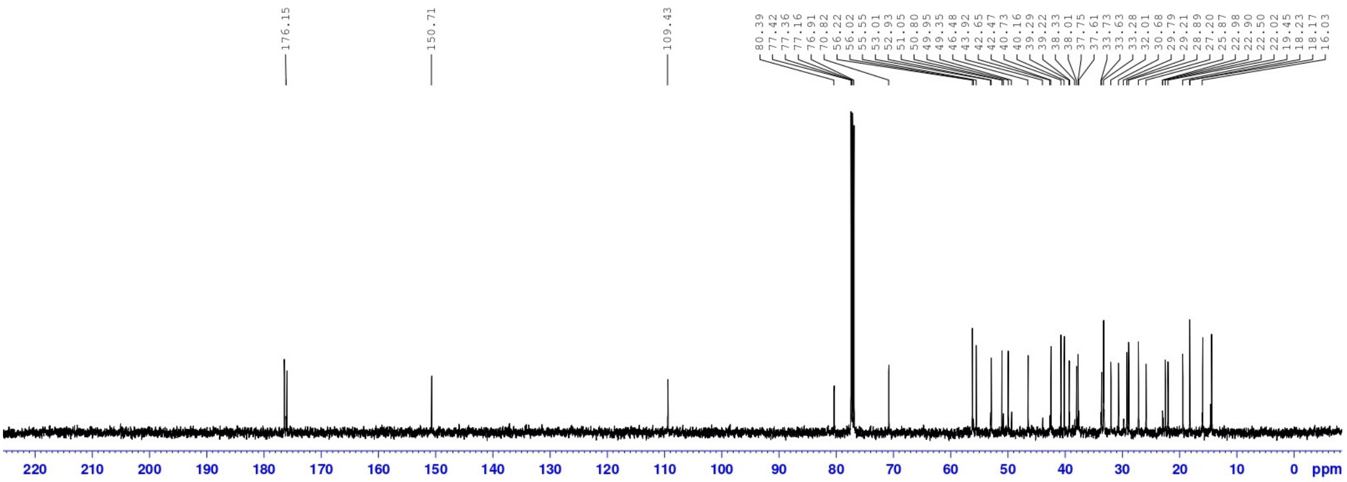

Figure S2. ^{13}C NMR spectrum of compound **20**

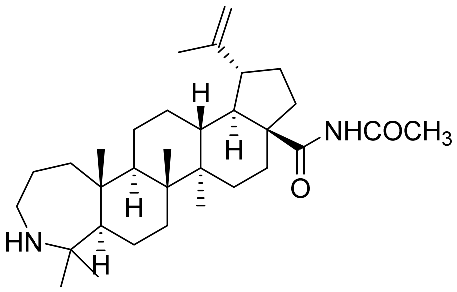

3-Deoxo-3a-homo-3a-aza-lup-20(29)-en-carboxamide acetate (**22**)

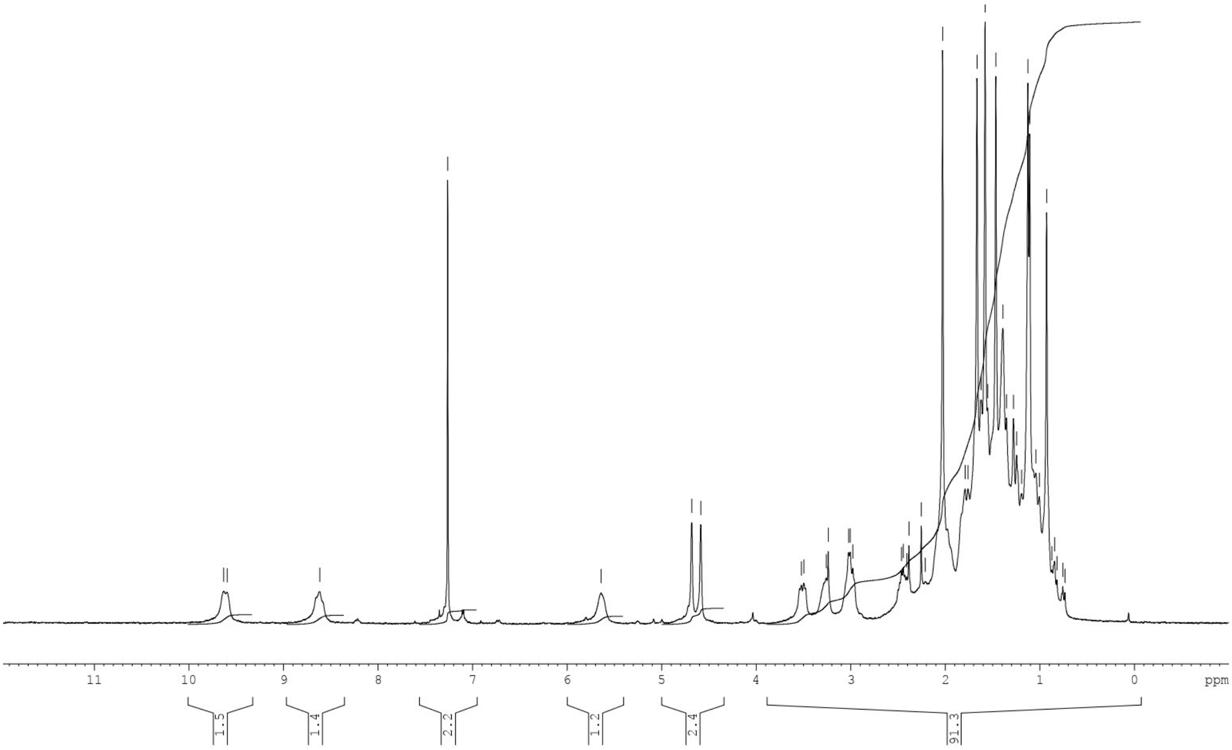

Figure S3. ^{1}H NMR spectrum of compound **22**

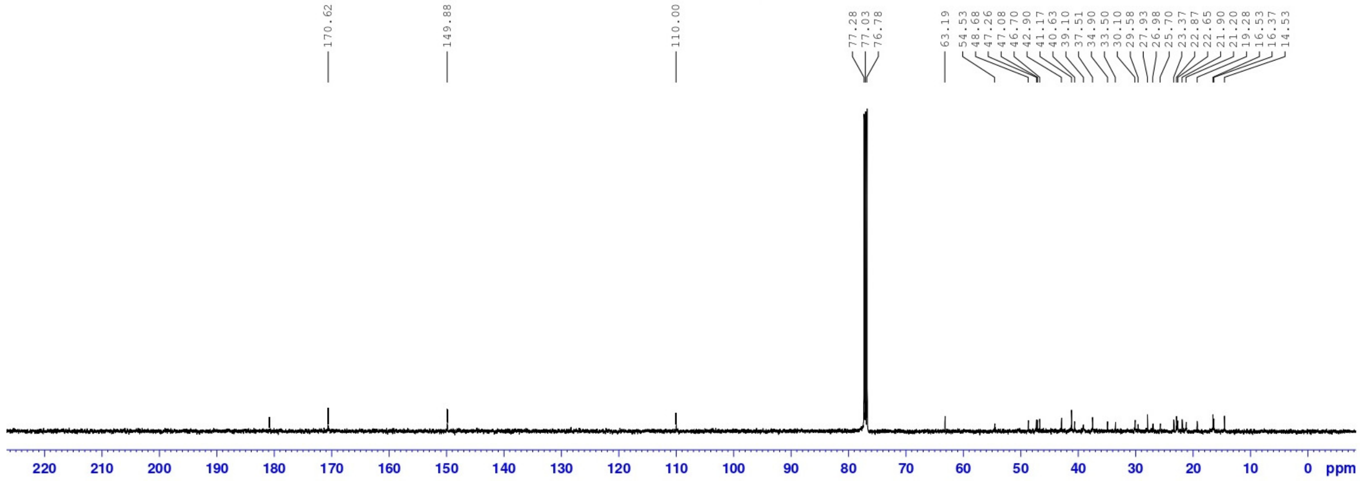

Figure S4. ^{13}C NMR spectrum of compound **22**

Table S1. Training data set for antiTB activity prediction – antiTB drugs

antiTB name	InChI key	DB ID
Aminosalicylic acid	WUBBRNOQWQTFEX-UHFFFAOYSA-N	DB00233
Cycloserine	DYDCUQKUCUHJBH-UWTATZPHSA-N	DB00260
Rifampicin	JQXXHWHPUNPDRT-WLSIYKJHSA-N	DB01045
Capreomycin	VCOPTHOUUNAYKQ-WBTCAYNUSA-N	DB00314
Ethionamide	AEOCXXJPGCBFJA-UHFFFAOYSA-N	DB00609
Ethambutol	AEUTYOVWOVBAKS-UWVGGRQHSA-N	DB00330
Bedaquiline	QUIJNHUBAXPXFS-XLJNKUFUSA-N	DB08903
Pretomanid	ZLHZLMOSPGACSZ-NSHDSACASA-N	DB05154
Viomycin	GXFAIFRPOKBQRV-GHXCTMGLSA-N	DB06827
Rifamycin	HJYYPODYNSCCOU-ODRIEIDWSA-N	DB11753
Rifabutin	ATEBXHFBFRCZMA-VXTBVIBXSA-N	DB00615
Rifapentine	WDZCUPBHRAEYDL-GZAUEHORSA-N	DB01201
Isoniazid	QRXWMOHMRWLFEY-UHFFFAOYSA-N	DB00951
Protionamide	VRDIULHPQTYCLN-UHFFFAOYSA-N	DB12667
Pyrazinamide	IPEHBUMCGVEMRF-UHFFFAOYSA-N	DB00339
Terizidone	ODKYYBOHSVLGNU-IAGONARPSA-N	DB12954
Delamanid	XDAOLTSRNUSPPH-XMMPIXPASA-N	DB11637
Tiocarlide	BWBONKHPVHMQHE-UHFFFAOYSA-N	DB13608
Enviomycin	HPWIIERXAFODPP-GHBBWTPBSA-N	DB08993
Morinamide	GVTLAVKAVSKBKK-UHFFFAOYSA-N	DB13417
Streptomycin	UCSJYZPVAKXKNQ-HZYVHMACSA-N	DB01082

Table S2. Training data set for antiTB activity prediction – non-antiTB drugs

Name	InChI key	DB ID
Metformin	XZWYZXLIPXDOLR-UHFFFAOYSA-N	DB00331
Tolbutamide	JLRGJRBPOGGCBT-UHFFFAOYSA-N	DB01124
Glimepiride	WIGIZIANZCJQQY-RUCARUNLSA-N	DB00222
Pioglitazone	HYAFETHFCAUJAY-UHFFFAOYSA-N	DB01132
Sitagliptin	MFFMDFFZMYYVKS-SECBINFHSA-N	DB01261
Canagliflozin	XTNGUQKDFGDXSJ-ZXGKGEBGSA-N	DB08907
Oxandrolone	QSLJIVKCVHQPLV-PEMPUTJUSA-N	DB00621
Ascorbic acid	CIWBSHSKHKDKBQ-JLAZNSOCSA-N	DB00126
Benfotiamine	BTNNPSLJPBRMLZ-LGMDPLHJSA-N	DB11748
Vitamin A	FPIPGXGPPPQFEQ-OVSJKPMPSA-N	DB00162
Betaine	KWIUHFFTVRNATP-UHFFFAOYSA-N	DB06756
Lorcaserin	XTTZERNUQAFMOF-QMMMGPOBSA-N	DB04871
Ephedrine	KWGRBVOPPLSCSI-WPRPVWTQSA-N	DB01364
Mesalazine	KBOPZPXVLCULAV-UHFFFAOYSA-N	DB00244
Loperamide	RDOIQAHITMMDAJ-UHFFFAOYSA-N	DB00836
Morphine	BQJCRHHNABKAKU-KBQPJGBKSA-N	DB00295
Miconazole	BYBLEWFAAKGYCD-UHFFFAOYSA-N	DB01110

Phthalylsulfathiazole	PBMSWVPMRUJMPE-UHFFFAOYSA-N	DB13248
Sorbitol	FBPFZTCFMRRESA-JGWLITMVSA-N	DB01638
Lactitol	VQHSOMBJVWLPSR-JVCRWLNRSA-N	DB12942
Bisacodyl	KHOITXIGCFIULA-UHFFFAOYSA-N	DB09020
Glycyrrhizic acid	LPLVUJXQOOQHMX-QWBHMCJMSA-N	DB13751
Ursodeoxycholic acid	RUDATBOHQWOJDD-UZVSRGJWSA-N	DB01586
Ondansetron	FELGMEQIXOGIFQ-UHFFFAOYSA-N	DB00904
Aprepitant	ATALOFNDEOCMKK-OITMNORJSA-N	DB00673
Domperidone	FGXWKSZFVQUSTL-UHFFFAOYSA-N	DB01184
Atropine	RKUNBYITZUJHSG-SPUOUPEWSA-N	DB00572
Drotaverine	OMFNSKIUKYOYRG-MOSHPQCFSA-N	DB06751
Methantheline	GZHFODJQISUKAY-UHFFFAOYSA-N	DB00940
Oxyphencyclimine	DUDKAZCAISNGQN-UHFFFAOYSA-N	DB00383
Lansoprazole	MJIHNNLFOKEZEW-UHFFFAOYSA-N	DB00448
Misoprostol	OJLOPKGSLYJEMD-URPKTTJQSA-N	DB00929
Nizatidine	SGXXNSQHWDMGGP-UHFFFAOYSA-N	DB00585
Tromethamine	LENZDBCJOHFCAS-UHFFFAOYSA-N	DB03754
Folic acid	OVBPIULPVIDEAO-LBPRGKRZSA-N	DB00158
Fostamatinib	GKDRMWXFWHEQQT-UHFFFAOYSA-N	DB12010
Phylloquinone	MBWXNTAXLNYFJB-NKFFZRIASA-N	DB01022
Tranexamic acid	GYDJEQRTZSCIOI-LJGSYFOKSA-N	DB00302
Prasugrel	DTGLZDAWLRGWQN-UHFFFAOYSA-N	DB06209
Warfarin	PJVWKTKQMONHTI-UHFFFAOYSA-N	DB00682
Acipimox	DJQOOSBJCLSSEY-UHFFFAOYSA-N	DB09055
Bezafibrate	IIBYAHWJQTYFKB-UHFFFAOYSA-N	DB01393
Atorvastatin	XUKUURHRXDUEBC-KAYWLYCHSA-N	DB01076
Aliskiren	UXOWGYHJODZGMF-QORCZRPOSA-N	DB09026
Valsartan	ACWBQPMHZXGDFX-QFIPXVFZSA-N	DB00177
Perindopril	IPVQLZZIHOAWMC-QXKUPLGCSA-N	DB00790
Diltiazem	HSUGRBWQSSZJOP-RTWAWAEBSA-N	DB00343
Lercanidipine	ZDXUKAKRHYTAKV-UHFFFAOYSA-N	DB00528
Bisoprolol	VHYCDWMUTMEGQY-UHFFFAOYSA-N	DB00612
Polidocanol	ONJQDTZCDSESIW-UHFFFAOYSA-N	DB06811
Nitroglycerin	SNIOPGDIGTZGOP-UHFFFAOYSA-N	DB00727
Lidocaine	NNJVILVZKWQKPM-UHFFFAOYSA-N	DB00281
Dexamethasone	UREBDLICKHMUKA-CXSFZGCWSA-N	DB01234
Dihydroergocristine	DEQITUUQPICUMR-HJPBWRTMSA-N	DB13345
Spironolactone	LXMSZDCAJNLERA-ZHYRCANASA-N	DB00421
Etacrynic acid	AVOLMBLBETYQHX-UHFFFAOYSA-N	DB00903
Mecamylamine	IMYZQPCYWPFTAG-UHFFFAOYSA-N	DB00657
Mexiletine	VLPIATFUUWWMKC-UHFFFAOYSA-N	DB00379
Digoxin	LTMHDMANZUZIPE-PUGKRICDSA-N	DB00390
Benzoyl peroxide	OMPJBNCRMGITSC-UHFFFAOYSA-N	DB09096
Cyclosporine	PMATZTZNYRCHOR-CGLBZJNRSA-N	DB00091
Dimethyl fumarate	LDCRTTXIJACKKU-ONEGZZNKSA-N	DB08908
Lomustine	GQYIWUVLTXOXAJ-UHFFFAOYSA-N	DB01206

Chlorzoxazone	TZFWDZFKRBELIQ-UHFFFAOYSA-N	DB00356
Celecoxib	RZEKVGVHFLEQIL-UHFFFAOYSA-N	DB00482
Fluvoxamine	CJOFXWAVKWHTFT-XSFVSMFZSA-N	DB00176
Ethosuximide	HAPOVYFOVVWLRS-UHFFFAOYSA-N	DB00593
Praziquantel	FSVJFNAIGNNGKK-UHFFFAOYSA-N	DB01058
Ivermectin	SPBDXSGPUHCETR-CVSKBELMSA-N	DB00602
Fenoterol	LSLYOANBFKQKPT-UHFFFAOYSA-N	DB01288
Brinzolamide	HCRKCZRJWPKOAR-JTQLQIEISA-N	DB01194

Table S3. Lupane derivatives – antiTB drug mutual information complete results

	Aminosalicylic acid	Cycloserine	Rifampicin	Capreomycin	Ethionamide	Ethambutol	Bedaquiline	Pretomanid	Rifamycin	Rifabutin	Rifapentine	Isoniazid	Protionamide	Pyrazinamide	Terizidone	Delamanid	Tiocarlide	Enviomycin	Morinamide	Streptomycin	Average	Most Similar Drug1	Second Most Similar Drug	Third Most Similar Drug
**20**	1.0085	1.0516	1.0782	1.0049	0.9819	1.0415	0.9884	0.8973	1.049	1.015	1.0782	1.085	11.047	1.0616	1.0847	0.9518	1.0516	1.0516	0.9352	1.025	0.9726	Protionamide	Terizidone	Rifampicin
**3**	1.0185	1.0085	1.0616	0.9884	0.9919	1.025	0.9718	0.8807	0.9884	0.9718	1.0616	1.0185	1.0616	1.0451	1.015	0.9352	1.0085	1.0085	0.9187	1.0085	0.9518	Rifampicin	Protionamide	Rifapentine
**1**	0.9919	1.0085	1.0616	1.015	0.9653	1.025	0.9453	0.8542	0.9884	0.9718	1.0616	0.9919	1.0616	1.0716	1.015	0.9352	1.0085	1.0085	0.9187	1.0085	0.948	Pyrazinamide	Rifampicin	Protionamide
**13**	0.9984	0.9884	1.0415	0.9683	0.9718	1.0315	0.9784	0.8607	0.9683	0.9784	1.0415	0.9984	1.0681	1.0516	1.0215	0.9152	1.0415	1.0415	0.9252	1.015	0.9479	Protionamide	Pyrazinamide	Enviomycin
**9**	0.9553	0.9718	1.025	0.9784	0.9287	0.9884	0.9352	0.8176	0.9784	0.9618	1.025	0.9553	1.025	1.0085	0.9784	0.9518	0.9718	0.9718	0.9087	0.9984	0.9207	Rifampicin	Protionamide	Rifapentine
**8**	0.9553	0.9984	1.025	0.9518	0.9553	0.9884	0.9087	0.8176	0.9518	0.9618	1.025	0.9553	1.025	1.0085	1.0315	0.8986	0.9718	0.9718	0.8821	0.9718	0.9169	Terizidone	Rifampicin	Protionamide
**17**	0.9553	0.9718	1.025	0.9518	0.9287	0.9884	0.9352	0.8707	0.9518	0.9618	1.025	0.9553	1.025	1.0085	0.9784	0.8986	0.9718	0.9718	0.9087	0.9718	0.9169	Rifampicin	Protionamide	Rifapentine
**6**	0.9453	0.9884	1.0415	0.9417	0.9187	0.9784	0.9252	0.8075	0.9683	0.9252	1.015	0.9453	1.015	1.025	0.9683	0.8886	0.9618	0.9618	0.872	0.9884	0.9086	Rifampicin	Pyrazinamide	Rifapentine
**14**	0.9388	0.9553	1.0085	0.9618	0.9122	0.9984	0.8921	0.8276	0.9352	0.9187	1.0085	0.9653	1.0085	0.9919	0.9884	0.8821	0.9553	0.9553	0.8921	0.9819	0.9037	Rifampicin	Protionamide	Rifapentine

**10**	0.9287	0.9453	1.025	0.9252	0.9021	0.9884	0.9087	0.8176	0.9518	0.9352	0.9984	0.9287	0.9984	1.0085	0.9518	0.9352	0.9453	0.9984	0.8555	0.9453	0.8997	Rifampicin	Pyrazinamide	Enviomycin
**18**	0.9884	0.9518	1.0049	0.9051	0.9087	0.9417	0.9152	0.7975	0.9583	0.8886	1.0049	0.9352	0.9784	0.9618	0.9583	0.852	0.9252	0.9518	0.8886	0.9884	0.8907	Rifampicin	Rifapentine	Streptomycin
**16**	0.9618	0.9252	1.0049	0.9051	0.9087	0.9417	0.862	0.7975	0.9583	0.9152	0.9784	0.9352	1.0049	0.9884	0.9317	0.9051	0.9784	0.9518	0.8886	0.9252	0.889	Rifampicin	Protionamide	Pyrazinamide
**5**	0.8986	0.9683	0.9949	0.9217	0.8986	0.9849	0.8786	0.7609	0.8951	0.9051	0.9683	0.9518	0.9949	0.9784	0.9217	0.8419	0.9417	0.9417	0.852	0.9417	0.8781	Rifampicin	Protionamide	Ethambutol
**7**	0.8921	0.9352	0.9618	0.8886	0.8921	0.9518	0.8455	0.7544	0.8886	0.8986	0.9618	0.8921	0.9618	0.9453	0.9417	0.862	0.9087	0.9087	0.9087	0.9087	0.8623	Rifampicin	Protionamide	Rifapentine
**12**	0.9087	0.8986	0.9784	0.9317	0.8555	0.9949	0.8354	0.7975	0.9051	0.8886	0.9784	0.8821	0.9518	0.9352	0.9051	0.852	0.9252	0.9252	0.8088	0.8986	0.8598	Ethambutol	Rifampicin	Rifapentine
**2**	0.9073	0.8973	0.9504	0.8772	0.8807	0.9138	0.8607	0.743	0.9038	0.8607	0.9504	0.9339	0.977	0.9339	0.967	0.8506	0.9504	0.9504	0.8341	0.8973	0.859	Protionamide	Terizidone	Enviomycin
**15**	0.8921	0.8821	0.9352	0.862	0.839	0.8986	0.8189	0.7544	0.8886	0.872	0.9352	0.8655	0.9352	0.9187	0.8886	0.8088	0.8821	0.9087	0.8189	0.8821	0.8327	Rifampicin	Protionamide	Rifapentine
**4**	0.839	0.9352	0.9352	0.862	0.8124	0.872	0.872	0.7644	0.862	0.8189	0.9352	0.8655	0.9352	0.8921	0.9152	0.862	0.8555	0.9187	0.8189	0.8555	0.8299	Cycloserine	Protionamide	Rifapentine
**22**	0.8455	0.8886	0.9417	0.8685	0.8189	0.9051	0.8254	0.7609	0.8419	0.8254	0.9152	0.8455	0.9152	0.8986	0.9317	0.8419	0.862	0.862	0.7722	0.9152	0.8229	Rifampicin	Terizidone	Streptomycin
**11**	0.8756	0.8655	0.9187	0.8455	0.8224	0.8821	0.8289	0.7378	0.8455	0.8555	0.9718	0.849	0.9187	0.9021	0.872	0.8189	0.8655	0.8921	0.8023	0.8921	0.822	Rifapentine	Rifampicin	Protionamide
